# Characterization of proteolytic degradation products of vaginally administered bovine lactoferrin

**DOI:** 10.1371/journal.pone.0268537

**Published:** 2022-05-19

**Authors:** Thomas P. Hopp, Klaudyna Spiewak, Maura-Ann H. Matthews, Zafeiria Athanasiou, Richard S. Blackmore, Gary A. Gelbfish

**Affiliations:** 1 Metrodora Therapeutics LLC, Brooklyn, New York, NY, United States of America; 2 Department of Surgery, Mount Sinai School of Medicine, New York, NY, United States of America; Fondazione Pisana per la Scienza, ITALY

## Abstract

When bovine lactoferrin (bLF) contacts human vaginal fluid (VF) it is subjected to proteolytic degradation. This report describes fragmentation patterns of bLF dosed vaginally in clinical trials or incubated *ex vivo* with VF. A consensus pattern of fragments was observed in samples from different women. The 80 kDa bLF molecule is initially cleaved between its homologous 40 kDa domains, the N-lobe and C-lobe, and then degraded into sub-fragments and mixtures of small peptides. We characterized this fragmentation process by polyacrylamide gel electrophoresis, western blotting, chromatographic separation, and mass spectral sequence analysis. Common to most VF fragmentation patterns were large amounts of an N-lobe 37 kDa fragment and a C-lobe 43 kDa fragment resulting from a single cleavage following tyrosine 324. Both fragments possessed full sets of iron-ligand amino acids and retained iron-binding ability. In some VF samples, alternative forms of large fragments were found, which like the 37+43 kDa pair, totaled 80 kDa. These included 58+22 kDa, 18+62 kDa, and 16+64 kDa forms. In general, the smaller component was from the N-lobe and the larger from the C-lobe. The 18+62 kDa pair was absent in some VF samples but highly abundant in others. This variability suggests multiple endopeptidases are involved, with the 18 kDa fragment’s presence dependent upon the balance of enzymes. Further action of VF endopeptidases produced smaller peptide fragments, and we found evidence that exopeptidases trimmed their N- and C-termini. The 3.1 kDa antimicrobial peptide lactoferricin B was not detected. These studies were facilitated by a novel technique we developed: tricolor western blots, which enabled simultaneous visualization of N- and C-terminal epitopes.

## Introduction

Bovine lactoferrin (bLF) is a milk protein with wide applications as a nutritional supplement, a food additive, and a medicine with a variety of potential uses in human and animal health. This multi-functional iron-binding protein of the transferrin family possesses antimicrobial properties, which depend on its strongly cationic nature and its iron-sequestering ability. bLF is a single chain, 80-kDa glycoprotein of 689 amino acids [1] comprised of two homologous, 40 kDa N- and C-terminal lobes, each containing an iron binding site with the capacity to reversibly bind one ferric ion (Fe^3+^) for a total of two irons per 80 kDa bLF molecule [2]. bLF is an important component of the innate immune system and is present in neutrophil granules, tears, saliva, and other mucosal secretions. Its bacteriostatic and bactericidal effects result both from its ability to bind iron and also from its lipopolysaccharide-binding properties. Furthermore, it contributes to maintaining healthy microbiomes in the gastrointestinal tract and vagina by being selectively bactericidal, sparing lactobacilli, bifidobacteria, and other commensal organisms, but killing pathogens [3–6].

Clinical and preclinical studies have been conducted with orally administered bLF and recombinant human lactoferrin in various indications that exploit lactoferrin’s iron-binding and LPS-binding properties. Wang [7] recently reviewed the many physiological effects of bLF, and its uses in medicines and dietary supplements. It has seen applications in dental care, appearing in mouthwashes and toothpastes, and in wound healing products [7]. bLF has also shown promise for prevention of late onset sepsis in low-birth-weight infants [8, 9] and is known to beneficially modulate the microbiome of the infant gut [10]. Other indications include treatment of gastric *H*. *pylori* infection [11, 12], pregnancy associated anemia [13–16], and Crohn’s disease. These therapies are administered orally and consequently the gastrointestinal metabolism of lactoferrin has been of great interest.

Proteolytic degradation of bLF by the stomach enzyme pepsin was studied by Troost [17] who showed that a major proportion (60–80%) of bLF administered in a high dose (15 mg/mL) survived passage through the stomach in adults, and that the transit time in the duodenum was 20 to 30 min after instillation. However, in a subsequent study in women with ileostomies, orally ingested human LF was totally degraded in the upper GI tract [17]. Furlund and colleagues [18] investigated *in vivo* gastric and duodenal digestion of bLF, reporting a great variety of fragments dependent on the pH and enzymes present in the digestion mixes.

Recently, bLF has been tested for its applicability to vaginal infections, and in particular, bacterial vaginosis (BV). Researchers at University of Catania [6] showed that vaginal suppositories containing bLF can improve BV and do so by shifting the vaginal microbiome from pathogen-dominated to normal flora.

Metrodora Therapeutics is conducting a Phase 1 clinical trial (ACTRN12619000295145) evaluating the safety and vaginal pharmacokinetics of multiple formulations of Metrodora Therapeutics bLF (MTbLF). While the results of the trial have not yet been published, this report characterizes bLF proteolytic fragments observed in VF specimens collected in that study. We also describe fragmentation patterns in controlled *ex vivo* digests using specimen-banked human VF samples. We further demonstrate retention of iron-binding capacity of several larger fragments which, along with whole MTbLF, persisted for up to 24 hr in VF. Thus, despite partial proteolytic degradation, vaginally dosed bLF retains sufficient iron-sequestering capacity to influence the makeup of the vaginal microbiome.

## Materials and methods

### MTbLF

MTbLF bovine lactoferrin was isolated from pasteurized skim milk using cation exchange chromatography and ultrafiltration techniques in a cGMP-compliant industrial-scale process, then formulated as tablets for vaginal administration. MTbLF is predominantly the bLF-b form, which has been noted in the literature as unglycosylated at asparagine 281, while the bLF-a form is highly glycosylated at that position [19]. Asparagines at 233, 368, 476, and 545 in MTbLF have been determined to be highly glycosylated with carbohydrate moieties that average 1,080 daltons each (unpublished). These carbohydrate moiety weights were used in calculating theoretical molecular weights of MTbLF fragments in polyacrylamide gel electrophoresis (PAGE) and mass spectrometry (MS) experiments.

### Vaginal suppository formulations

Suppository tablets were compacted from blended solid components including 300 mg of MTbLF, mannitol, and various gelling and mucoadhesive agents. Several similar formulations were investigated, all of which were able to release MTbLF slowly over a 2-to-24-hour timeframe.

### Vaginal fluid sample collection and storage

Clinical specimens were collected before and after vaginal administration of 300 mg MTbLF to premenopausal women between the ages 18 and 50. PVA Lasik Spears (eye surgery sponges) were used to absorb VF. Swabs were kept frozen at -20°C until processed. VF was isolated from swabs by centrifugation using Costar 0.45 μm Spin-X filters. VF was diluted with 4x sample buffer for SDS-PAGE or with 0.1% TFA for RP-HPLC analysis.

The trial from which the clinical specimens were collected received ethics approval from the Human Research and Ethics Committee of the Alfred Hospital, Melbourne, Australia (Project number 493/18). Written informed consent was obtained from all participants. In compliance with requirements of Alfred Hospital’s Ethics Committee, this study was prospectively filed with Australia’s Therapeutic Goods Administration via the Clinical Trial Notification scheme (CTN; clinical trial: CT-2018-CTN-03362-1) and registered with the Australian New Zealand Clinical Trials Registry (ANZCTR; ACTRN12619000295145).

### *Ex vivo* digestion of MTbLF in vaginal fluid

Vaginal fluid for *ex vivo* digests was purchased from Lee Biosolutions (catalogue 991-10-S) Maryland Heights, MO. These VF samples had been stored at -20°C without additives.

MTbLF was digested under controlled conditions by dissolving it at 20 mg/mL in 100 mM lactic acid, 150 mM NaCl buffer, pH 3.5 (adjusted with NaOH) and then combining 1:1 v/v with samples from the VF specimen bank. These mixtures were incubated at 37°C in sealed microfuge tubes, and time point aliquots were removed at 2, 4, and 24 h. The digestion reaction in each time-point sample was stopped by addition of 1/10th volume of 1 M tris hydroxide to bring the pH to neutral. These timed aliquots were either directly injected onto the S-200 SEC column or mixed with SDS PAGE sample buffer and heated at 90°C for 10 min for electrophoresis and western blotting procedures. Any excess was stored frozen at -20°C.

### Polyacrylamide gel electrophoresis

PAGE analysis was carried out using the NuPAGE system (Invitrogen) with 4–12% acrylamide precast gradient gels and Coomassie brilliant blue staining. Clinical samples already in PAGE buffer were further diluted 10-fold with 1x sample buffer before loading PAGE gel lanes. SeeBlue-Plus-2 pre-stained standard protein ladders (Invitrogen) were used in PAGE and Western experiments. Standard protein lanes were run on all gels, including either standard MTbLF, SeeBlue-Plus-2 standards, or both, so that each gel had at least one standard lane and sometimes several. These standard lanes were used to superimpose gel images so fragment sizes could be compared precisely, despite sometimes being run on separate gels. This was especially critical to allow the creation of tri-color PAGE / western images (described below and in the supplemental materials) by overlaying three separate gel images in precise register.

In total, VF samples from 13 clinical subjects and 9 VF donors were utilized in the experiments reported here. The number of time-point samples varied from subject to subject, but in total 891 individual polyacrylamide-gel lanes were obtained showing fragmentation patterns for bLF in VF. This PAGE/western data set included 52 gels of clinical samples (Au1-Au52) and 47 gels of specimen-bank VFs with bLF added *ex vivo* (L13-L60). At 12 lanes per gel, the full dataset included a total of 1,188 lanes, including standard lanes. Original PAGE and western images are available in supplement S1 Fig.

### Western blots

PAGE gels were transferred to nitrocellulose filters using the iBlot system (Invitrogen) and color was developed using Western-Breeze reagents, with the exception that the kit’s milk-based blocking solution was replaced with 1% w/v bovine serum albumin dissolved in the kit’s washing buffer, to avoid interference from bLF present in milk products. Alkaline phosphatase-conjugated goat-anti-mouse secondary antibodies and chromogenic substrate were kit components. Primary monoclonal antibodies (mAbs) were murine N-lobe-reactive anti-bovine lactoferricin B (GWB-C768F7) and anti-lactoferrin C-Lobe (GWB-1A2A49) from Genway.

### Tri-color images

Combined images of two western blots and one Coomassie-stained PAGE gel were used to generate tri-color PAGE/western images. Identical samples were run on each of three component gels, then developed with single reagents to show bands reacting with N-lobe, C-lobe, or Coomassie stain. The three gels were imaged in black-and-white, then superimposed using the following procedure in Photoshop.

The three black-and-white images were reversed to black backgrounds with the INVERT function of Photoshop, then colorized monochromatically to the complementary colors, cyan for red, magenta for green, and yellow for blue. These negative monochrome images were then overlayed as separate Photoshop layers with their TRANSPARENCY set to 0 for the yellow bottom layer, 50 for the magenta middle layer, and 70 for the cyan top layer. The three layers were then combined with the MERGE LAYERS function to yield tri-color negative photos, which were then switched back to red-green-blue tricolor images by a second application of the INVERT function. The result was combination of three differentially stained gels into one tri-color image. Details of the process are shown in supplement S2 Fig. To our knowledge, this method has not been reported prior to this paper.

### Size-exclusion (SEC) HPLC analysis

SEC analysis was performed on a Hewlett Packard 1050 HPLC system equipped with a TSKgel G2000SWxl column, 7.8 mm inner diameter, 30 cm length, and 5 μm particle size, equilibrated with 50 mM NaH_2_PO_4_, 300mM NaCl, 5 mM NaHCO_3_, pH 7.4 running buffer. Injection volume was 30 μL, flow rate was 0.6 mL/min, column temperature was 25°C. The column was separately calibrated using molecular weight standard proteins IgG (160 kDa), BSA (65 kDa), soybean trypsin inhibitor (21.5 kDa), and aprotinin (6.5 kDa). Elution profiles were monitored by absorbance at 280 and 466 nm to follow protein and lactoferrin-bound iron simultaneously.

### Reversed-phase HPLC analysis

Reversed-phase HPLC was used for separation and analysis of bLF and its vaginal-fluid fragments. A Hewlett Packard 1050 HPLC system with a reversed phase C3 column (RPC3; Zorbax 300SB-C3, 3.5 μm, 4.6 x 150 mm, Agilent #863973–909) equipped with a guard column (Zorbax 300SB-C3, 5μm, 4.6 x 12.5 mm (Agilent #820950–924) was operated at 1.0 mL/min and 25°C. Solvent A was 0.1% trifluoroacetic acid in water; solvent B was 0.01% trifluoroacetic acid in acetonitrile. A gradient from 10% to 70% B in 17 minutes was sufficient to separate bLF and its major fragments. Clinical VF samples were clarified by passage through Spin-X 0.45 μm centrifugal filters before applying to the HPLC.

### Mass spectrometry analysis

Two methods of MS analysis were used to characterize MTbLF fragmentation products. First, top-down MS was applied directly to the fragment mixtures generated by *ex vivo* digestion of bLF by VF. Second, to study isolated fragments, PAGE analysis plus tryptic peptide mapping was used to gather MS information on each fragment. When the linear sequence extent of each fragment was determined, it was cross-checked by comparing PAGE-determined molecular weights with theoretical calculations by the ExPASy mass-determination algorithm.

### Top-down MS analysis of bLF digest samples

MTbLF *ex vivo* VF digestion samples (0, 2, 4, and 24 hr time points) were analyzed without prior chromatography or other separation techniques to characterize the greatest possible number of fragments. The time-point samples were acidified with formic acid, clarified by centrifugation, and analyzed on a Dionex UltiMate 3000 RSLC nano system (ThermoFisher) operating in nanoflow mode coupled to an Orbitrap Fusion Lumos (ThermoFisher) mass spectrometer. The HPLC column was a 75 μm x 150 cm PepMap C4 column (ThermoFisher) using a 60 min gradient from 2 to 90% acetonitrile.

The mass spectrometer was operated in intact protein mode using 2–3 mTorr ion-routing multipole pressure. MS2 spectra were acquired using Orbitrap (OT) HCD or EThcD MS2 fragmentation modes with Top 3–5 DDA methods. OT MS1 data was acquired at resolution settings of 120K at m/z 200 and OTMS2 at a resolution of 120K at m/z 200. Precursor ion isolation was performed with the mass selecting quadrupole and the isolation window set to 3 m/z. The AGC target value was set to 5e5 for both MS1 and MS2; maximum injection times of 100 msec x 5 microscans for MS1 and 200–250 msec x 5 microscans for MS2 were used.

The top-down data were analyzed with Proteome Discoverer 2.4 utilizing the ProSightPD node, particularly the PSPD Subsequence Search node. All searches were performed against a database limited to the bLF sequence. Results were filtered using E value cutoff of 1×e-5 and search engine rank 1.

### PAGE and tryptic maps for MS

In the second MS method, bands were cut from SDS-PAGE gels, subjected to trypsinization, then analyzed by MS. Briefly, the excised and diced gel bands were destained with 50% acetonitrile in 25 mM NH_4_HCO_3_ for 15 min, reduced with 15 mM TCEP in 25 mM NH_4_HCO_3_ at room temp for 10 min, alkylated with 25 mM iodoacetamide in 25 mM NH_4_HCO_3_ (room temp for 45 min), washed once with 50% acetonitrile in 25 mM NH_4_HCO_3_ for 15 min, dehydrated with 100% acetonitrile and finally allowed to air dry for approximately 5 min. Protein gel samples were rehydrated with trypsin solution (2.5 ng/μL) containing 0.01% ProteaseMAX Surfactant (Promega) then incubated for 1 hour at 50°C. Each digestion supernatant was aspirated into a clean tube, then the gel pieces were extracted with 60% acetonitrile/1% trifluoroacetic acid to recover more peptides. The two portions were combined, dried, then cleaned up by resuspending in 10 μL 5% acetonitrile/0.1% trifluoroacetic acid and adsorption to a C18 tip (10 μL, Pierce), washed with 10 μL 5% acetonitrile/0.1% trifluoroacetic acid and eluted with 10 μL 50% acetonitrile/0.1% formic acid. Each sample (5 μL) was analyzed by LC-MS using a Dionex RSLC nano HPLC coupled to an Orbitrap Fusion Lumos (ThermoFisher) mass spectrometer. Peptides were resolved using a 75 μm x 50 cm PepMap C18 column (ThermoFisher) with a 2 hr gradient from 2 to 90% acetonitrile in 0.1% formic acid.

For data analysis, all MS2 spectra were analyzed using Mascot (Matrix Science, London, UK; version 2.5.1.0). Mascot was set up to search against only bLF sequences. The digestion enzyme was set as trypsin or non-specific. Mascot was searched with a fragment ion mass tolerance of 0.60 Da and a parent ion tolerance of 10 ppm. Oxidation of methionine and carbamidomethylation of cysteine were specified in Mascot as variable modifications. Scaffold version 4.8.2 (Proteome Software Inc., Portland, OR) was used to validate MS2 based peptide and protein identifications. Peptide identifications were accepted if they were scored less than 1% false discovery rate by the Peptide Prophet algorithm with Scaffold delta-mass correction.

### Other chemicals and supplies

Other materials were generally reagent grade and/or cell culture compatible.

## Results

### bLF fragmentation patterns in clinical samples

**Fig 1:**
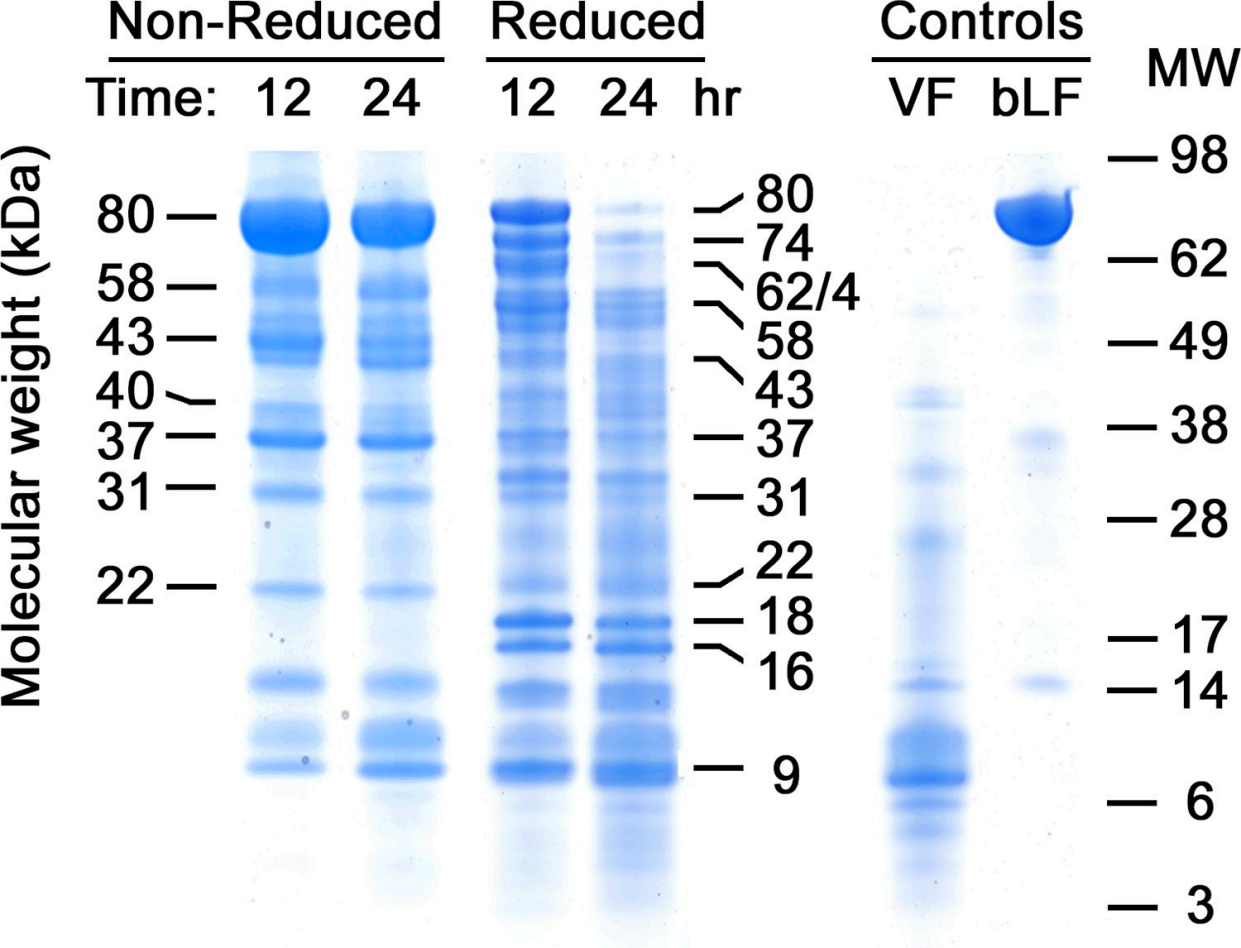
Fragmentation of bLF in human vaginal fluid. Characteristic patterns of fragments developed when MTbLF was dosed intravaginally in an individual clinical subject and followed over time. On the left, samples from two time points, 12 and 24 hr post dose, are shown without reduction, preserving disulfide bonds. Identical samples are shown in the next two lanes, after reduction to break disulfide bonds revealing nicked fragments present in the 80 kDa forms on the left. At right, control samples are shown, including the subject’s VF with no bLF present and intact MTbLF before dosing, in their reduced forms. The numbers on the left and mid-right identify the molecular weights of fragments that we repeatedly detected in VF samples from many, but not all, clinical subjects dosed with MTbLF. The bLF control lane was loaded with 5 μg of MTbLF. Numbers at far right represent SeeBlue-Plus-2 standard proteins run on the same PAGE gels as the samples.

The vaginal pharmacokinetics of lactoferrin suppository tablet formulations are being assessed in a phase 1 clinical trial in healthy women and women with BV (ACTRN12619000295145, manuscript in preparation). Vaginal fluid specimens from the trial were analyzed by SDS-PAGE to reveal patterns of proteolytic fragmentation. Fig 1 shows fragments present at 12 and 24 hr post dose for one participant. The fragmentation pattern shown is typical of many, though not all, subjects. Importantly, the fragment pattern is stable over time and whole 80 kDa bLF persists or is replenished throughout most of the time course as the solid lactoferrin tablet dissolves in VF. This suggests that the slow-release tablet provides a continuous source of 80 kDa material, which in turn replenishes the fragments for up to 24 hr in this case. Subsequently, all bLF-related bands disappear simultaneously, suggesting that once the dose has completely dissolved, the natural flushing action of fluid from cervix to introitus carries away any remaining whole bLF along with its fragments.

Fig 1 shows important characteristics of VF-proteolyzed MTbLF. First, one of the most striking differences between the non-reduced and reduced samples is the continued presence of a large amount of 80 kDa bLF in the non-reduced samples, while the 80 kDa form diminishes to trace amounts at 24 hr while the non-reduced band at 80 kDa remains strong. One interpretation of this dichotomy at 80 kDa is that proteolytic clips within 80 kDa bLF create a “nicked” molecule with fragments still held together by disulfide bonds, which are released into component parts when reduction breaks the disulfides. Not only does nicking occur within 80 kDa bLF, but it also occurs in several fragments, as we will show in detail below for the 43 kDa fragment.

Second, a limited number of distinct bands appear in either the non-reduced or reduced patterns. This suggests a limited number of proteases are responsible for cleavages that produce the fragments. Otherwise, a general streak might appear in each lane if multiple proteases were producing a great variety of degradation products. Notably, the number of bands is less for non-reduced samples than for reduced samples. This implies that the 17 disulfide bonds of native bLF hold together multiple proteolytic fragments in covalent groups that travel together during electrophoresis. Correspondingly, when reduced, these polypeptides are unlinked and separate to their own positions, resulting in a greater number of lower molecular weight bands in the reduced samples. As mentioned, most bands whether reduced or non-reduced, persist throughout the course of a dose. This contrasts with *in vitro* proteolytic digests, in which the high molecular weight bands disappear over time while low molecular weight bands arise as the digest exhausts the original whole protein in a typical substrate-product relationship. In these clinical samples however, resupply of whole bLF from the dissolving suppository provided a continuous source of 80 kDa protein, which yielded all fragments until the dose was fully dissolved after 24 hr and the flushing action of VF cleared all bands simultaneously.

Another phenomenon seen in Fig 1 is the clearer background in the non-reduced lanes and darker background in the reduced lanes, especially in the low molecular weight region. This diffuse staining may result from additional proteolytic processing of fragments into slightly smaller products by aminopeptidases, carboxypeptidases, or other exopeptidases, yielding continuous series of single- or double-amino acid shortenings of the main fragments. Note also that the 24-hr lane has more background in both the non-reduced and reduced samples, again consistent with exopeptidase trimming of fragments, whether disulfide bonded or not.

Also noteworthy in the reduced samples is a doublet pair of bands at 16 and 18 kDa. One or both bands often occur prominently in VF-degraded bLF, and due to their position where the background is usually clear, they are easily detectable and quantifiable products of proteolysis, whereas bands above and below them tend to overlap with other bands of similar molecular weight. Furthermore, in some VF samples, the 18 kDa component may be entirely absent (examples will be seen in several figures below) while the 16 kDa component tends to be strongly present in VF samples from all human subjects. The reason for this is not yet clear.

### Western analysis of the fragmentation pattern

**Fig 2:**
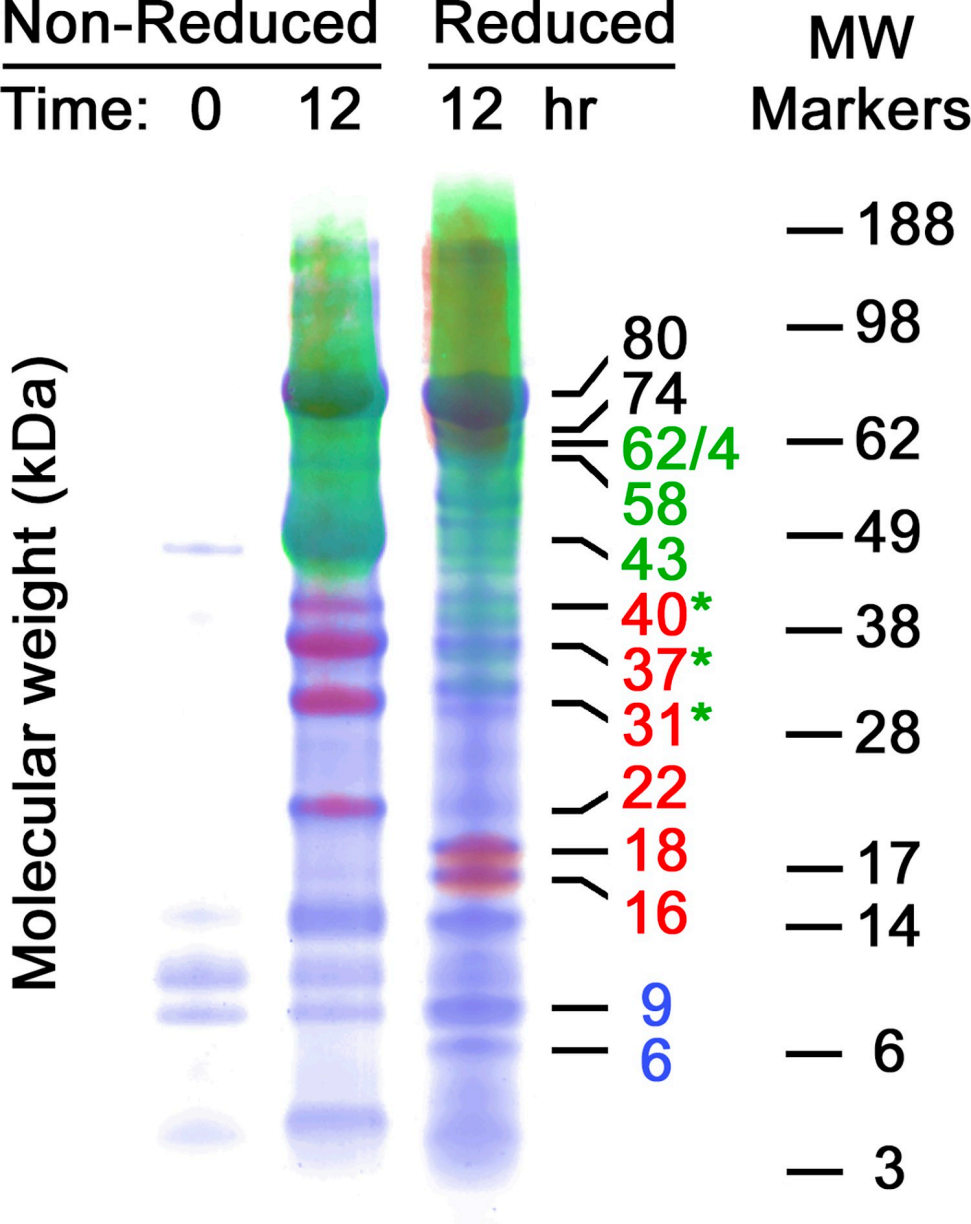
Tricolor image of PAGE gel lanes overlaid with two westerns. Selected time-point VF samples are shown for a second MTbLF-dosed clinical subject. To prepare this tricolor image, the three gels (Coomassie blue plus two mAb westerns) were put together in register by superimposing their SeeBlue standard ladders (weights shown at right), to match the gels precisely with each other. The molecular weight numbering scheme at middle right is colorized to indicate the major fragments along with their staining characteristics of red, green, or blue. Black numbers at 80 and 74 kDa indicate that those bands stained with all three stains. Green asterisks indicate the region where the red non-reduced bands disappear upon reduction and new, faint green bands appear (more on these green bands below). Blue numbers indicate bands that stained only with Coomassie blue, and hence lacked either epitope. Details of this new method of tricolor western imaging are given in the methods section and supplement S2 Fig.

Given the complexity of the band patterns, we sought to learn more about the fragments using western blot analysis with two mAbs, one specific for the lactoferricin B epitope near the N-terminus, and one for a second epitope within the C-lobe of bLF. Fig 2 shows the fragment patterns obtained in VF samples from a different clinical-trial participant. Overall, the band patterns are similar to those obtained with the first subject. As was seen for 80 kDa bLF in Fig 1, the 43 kDa component in this sample is probably nicked internally because it disappears for the most part in the reduced lane next to it. In contrast, the 80 kDa component does not show the same non-reduced/reduced dichotomy seen in Fig 1, perhaps because release of additional bLF from the tablet has replenished the 80 kDa band. Such variations in band intensities were commonly observed, presumably due to physiological differences between clinical subjects, and in single subjects during the course of treatment. Nevertheless, the band patterns in these two figures represent a common set of molecular-weight species seen almost universally in clinical samples.

Fig 2 also demonstrates a new way to present data when two mAbs are used. Briefly, three PAGE gels were run with identical samples, then stained with individual mAbs or Coomassie blue, and superimposed by Photoshop image-processing. In the example shown, blue color is from the Coomassie blue stained gel, red from the N-lobe antibody, and green from the C-lobe antibody. This technique provides a convenient, synoptic visualization of bands that contain one of the antigenic sites, both, or neither.

### Preparative *ex vivo* digestion of bLF

**Fig 3:**
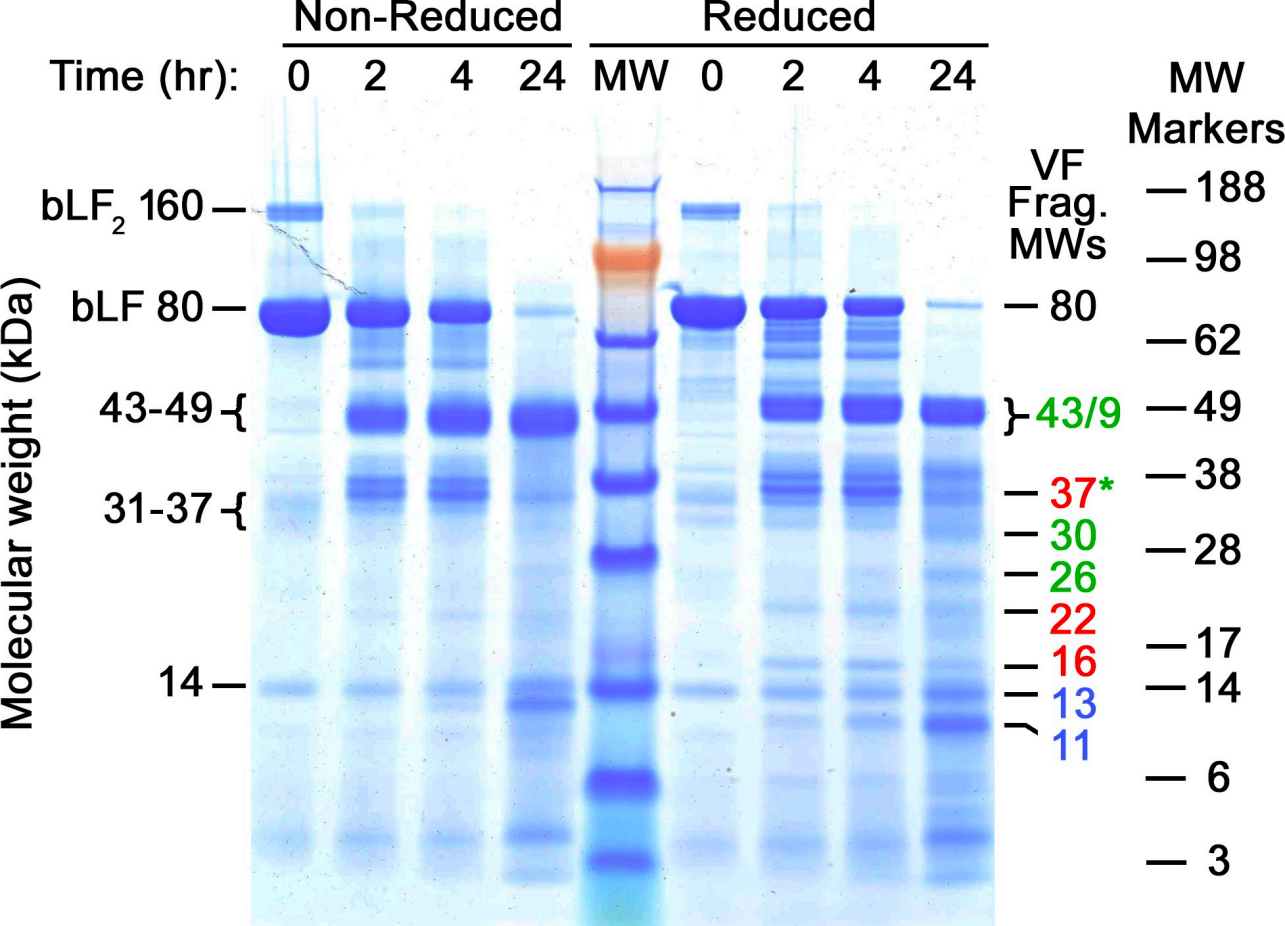
PAGE analysis of *ex vivo* VF digested MTbLF. Coomassie blue stained PAGE gel. Bands present in the original MTbLF are labeled on the left. Fragment molecular weight numbers are shown in the same color scheme as in Fig 2. The standard protein labels at far right correspond to the SeeBlue-Plus-2 standard ladder in the center lane (MW). The fragment bands obtained in this experiment were mostly identical to bands in Fig 2.

To further study the nature of the fragments, we worked with *ex vivo* digests of MTbLF in VF purchased from a specimen bank. One of these digests was subjected to PAGE to compare with the *in vivo* digestion patterns and later used for structural characterization work. Fig 3 shows many similarities to Figs 1 and 2, but also some differences.

Most notably, there is little evidence of nicking, which would appear as differences between the reduced and non-reduced lanes. However, the major bands at all time points look similar to those in Figs 1 and 2. The lack of nicking is surprising, given that most clinical samples show at least some evidence of the phenomenon. The absence of nicks may relate to the fact that these bands are products of a carefully controlled *in vitro* digestion at 37°C with a set pH of 3.5, while human clinical MTbLF samples were subject to natural variations in pH and other properties of VF *in vivo*. Nevertheless, the patterns shown in Fig 3 demonstrate excellent yields of bands in the 30-to-50 kDa region that were useful in the iron-binding and mass spectroscopy work described below.

There are two significant discrepancies between the bands in Figs 1 and 2, and those seen here. Most notable is the absence of the 18 kDa band present in those figures. This band appeared in more than half of all VF samples from our clinical study (manuscript in preparation) but is not present here. The second discrepancy is the relative lack of the 31 kDa band that is prominent in Figs 1 and 2. It would be oversimplifying to state that the lack of these bands reflects a fundamental difference between *ex vivo* and *in vivo* proteolysis, because some of our clinical samples also lack the 31- and 18-kDa bands. Regardless, this *ex vivo* digest provided substantial amounts of several important fragments for further characterization, described below.

### Iron binding by fragmentation products

**Fig 4:**
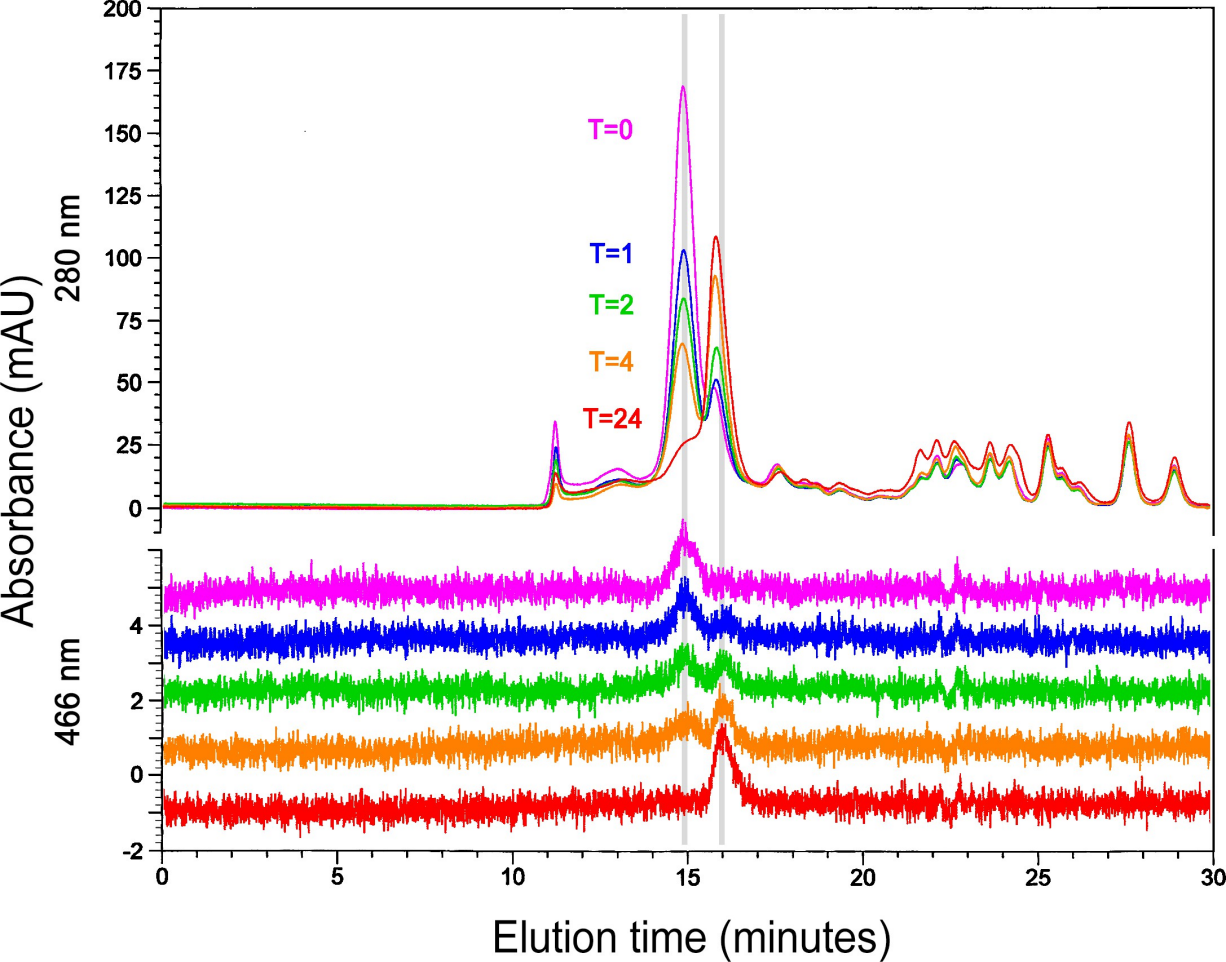
SEC HPLC of *ex vivo* digested MTbLF. Both the 280 nm traces (above) and the 466 nm traces (below) are shown. Color codes show the time points of the digestion samples. The 280 nm profiles are superimposed while the 466 nm profiles are separated for easier comparisons. The two vertical gray bars indicate the elution positions of 80 kDa bLF (~15 min) and of the ~40 kDa peak (~16 min). 160 kDa bLF dimer elutes to the left of the main peak, under the time point numbers. The spiked peak to the left of dimer is the excluded volume of the column, where bLF trimer and higher polymers were expected to elute. The total volume of the column occurs at 22 minutes. Materials eluting later are present at T = 0 and therefore are likely to be VF peptides or other non-bLF substances which bind to the column packing material.

To investigate whether any of the *ex vivo* fragments of bLF seen in Fig 3 retained the ability to bind iron we subjected those digestion timepoints to size exclusion HPLC chromatography on TSKgel G2000SW and measured iron binding at the 466 nm absorbance peak for Fe-lactoferrin [20, 21]. In addition to the main 80 kDa peak, we detected a separate later-eluting peak, which possessed 466 nm absorbance as well (Fig 4). This peak eluted at a position corresponding to approximately 40 kDa, which would include the N- and C-lobe fragments of 37 and 43 kDa, each of which contains a full set of iron ligating amino acids. Fig 4 compares the digestion time points, showing a clear correlation between the ~40 kDa peak rising in concert with the decreasing height of the 80 kDa bLF peak. Comparison with Fig 3 confirms the disappearance of the 80 kDa band in agreement with the peak height decrease on SEC HPLC, and also confirms that the 43 and 37 kDa bands rise simultaneously with the ~40 kDa peak on SEC HPLC.

### Preparative HPLC separation of clinical sample fragments

**Fig 5:**
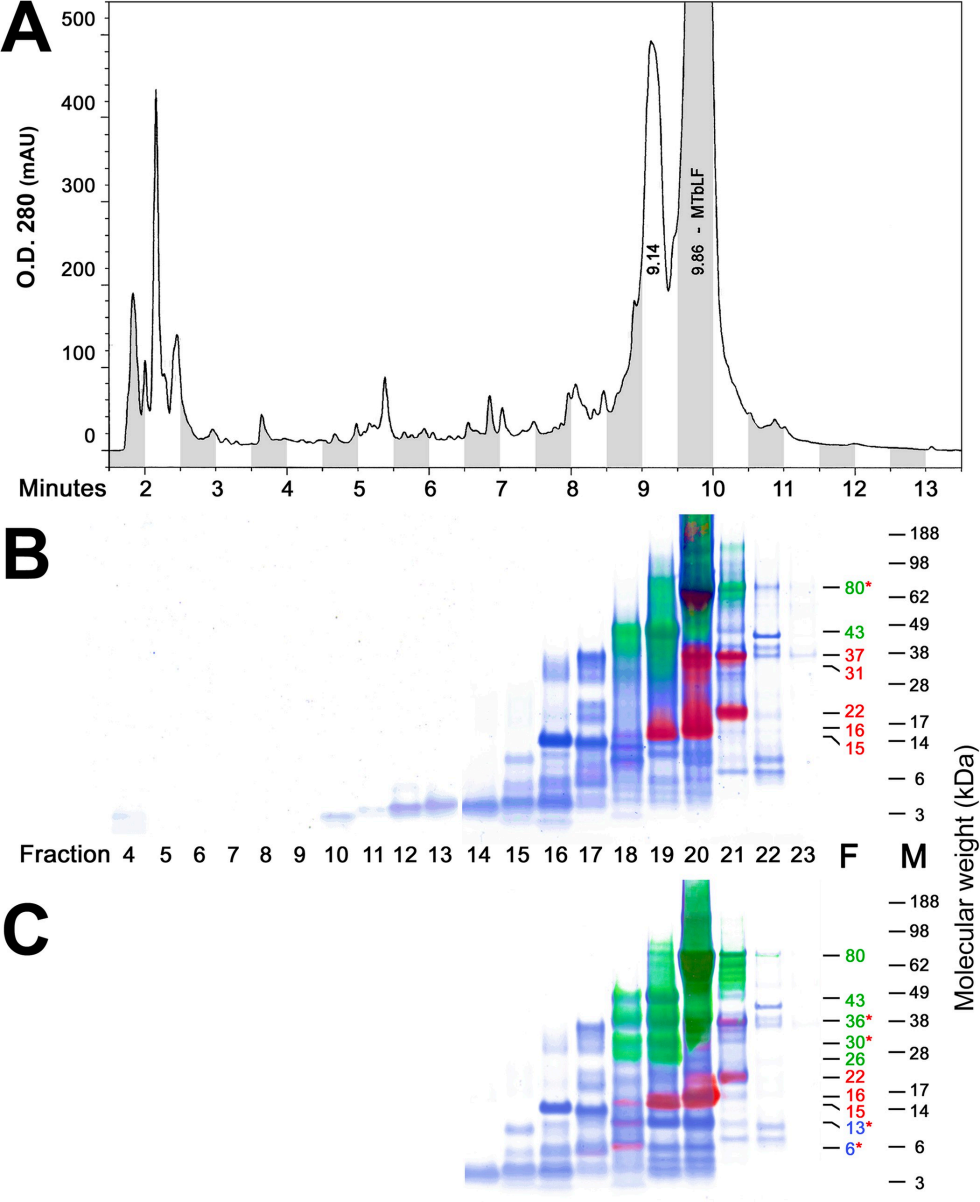
RPC3 HPLC of MTbLF-dosed VF with non-reduced and reduced tricolor westerns. The HPLC result in panel A shows a major peak at ~10 min for 80-kDa bLF and many earlier-eluting peaks corresponding to fragmented forms of bLF. In panels B and C, tricolor westerns of the HPLC fractions show many bands reactive with either the N-Lobe mAb (red) or the C-Lobe mAb (green), or non-epitope bearing but reactive with Coomassie stain (blue). Panel B shows samples electrophoresed without reduction, while Panel C shows the same samples after disulfide bonds were broken by reduction. Fragment molecular-weight numbers are shown, color-coded, at right. Asterisks indicate cases where separate peptides of the same molecular weight stain with different developing reagents in different lanes.

Clinical VF samples show characteristic patterns on RPC3 HPLC at different time points after dosing. Early time-point chromatograms showed only a single major peak of 80 kDa bLF eluting at approximately 10 minutes, plus many small peaks due to low levels of polypeptides normally present in VF. However, as Fig 5A shows, additional peaks appear at later time points, including a major one at about 9.1 minutes. This latter peak appears simultaneously with the ~40 kDa peak seen on SEC HPLC in Fig 4. Also similar to Fig 4, this 9.1-minute peak rises as the bLF peak falls. In Fig 5, VF samples from a single MTbLF-dosed clinical subject at 4–8- and 12-hour time points were pooled and subjected to preparative RPC3 HPLC, and eluate fractions were collected. Panels 5B and 5C show these fractions on tricolor PAGE/western gels, with disulfide bonds intact (5B), or reduced (5C).

**Fig 6:**
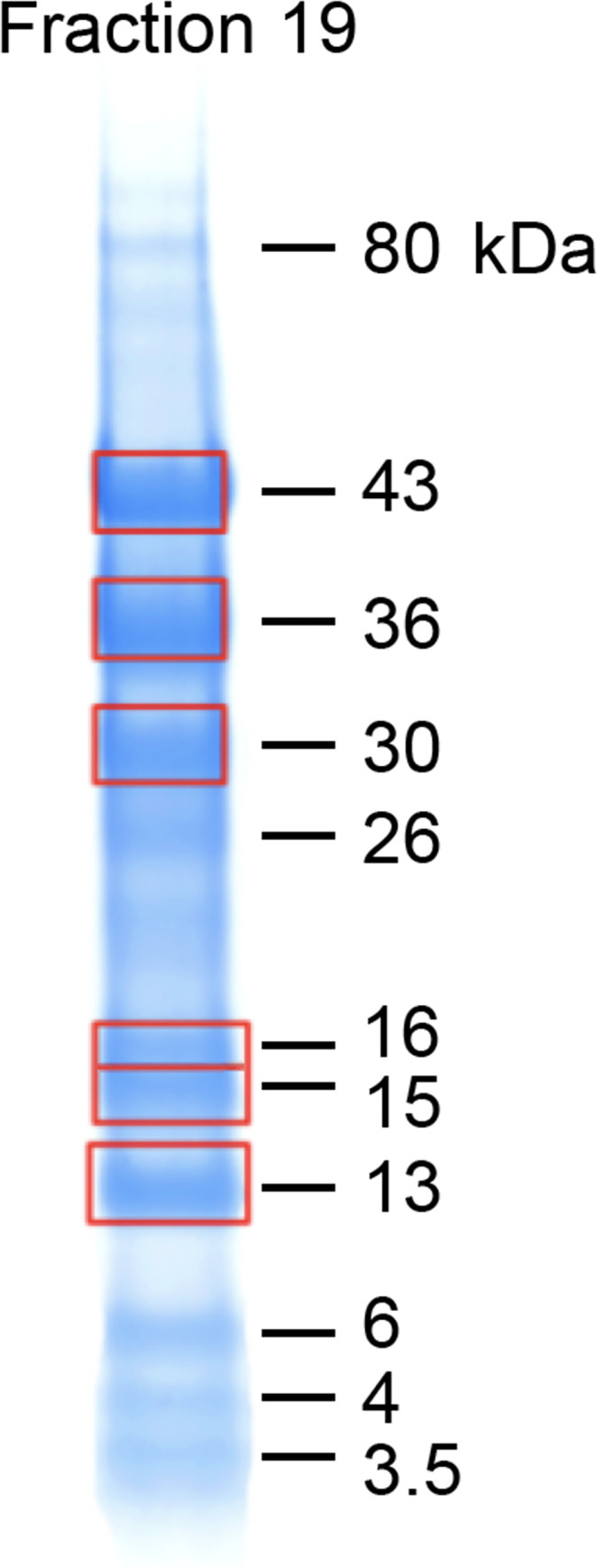
Excision of fraction 19 PAGE bands for trypsinization and MS. Fraction 19 of Fig 5C is shown with Coomassie blue staining only. Red boxes indicate bands excised from the gel, minced, destained, and trypsinized. Each trypsinized band was then subjected to LC-MS peptide mapping analysis.

In analyzing this complex chromatogram, we concentrated on Fraction 19, where the 43 kDa fragment was well separated from other high-molecular weight components. Also of interest were several sub-fragments seen after reduction (Panel C, Fraction 19), with molecular weights of 36, 30, and 26 kDa. Fig 6 shows the details of Fraction 19, Panel C, with Coomassie blue staining only, and boxes indicating the bands excised from the gel for tryptic digestion and MS identification of sub-fragments, as discussed below.

## Mass spectrometry findings

### Tryptic peptide mapping MS of PAGE separated fragments

Each of the six bands excised from the gel in Fig 6 were subjected to in-gel tryptic digestion and peptide mapping MS. LC-MS runs yielded mass matches to many of the expected tryptic fragments from throughout the molecule, especially within the C-lobe region. As expected, no peptides were detected bearing N-linked carbohydrate moieties, because the heterogeneity of carbohydrate moieties (from 2 to 11 sugar monomers attached at each position) divided the mass spectral signals and allowed no matches to calculated non-glycosylated polypeptide masses. On the other hand, many other tryptic peptides were detected and provided evidence concerning the extent most of the fragments. Once locations of the fragments were determined, it was possible to add back the undetected glycopeptide masses and then calculate total masses for the fragments. In doing this, an average carbohydrate mass of 1,080 daltons was used for the four glycosylation sites at asparagine residues 233, 368, 476, and 545 (as mentioned, asparagine 281 lacks a carbohydrate moiety).

### Information from peptide mass detection frequencies

**Fig 7:**
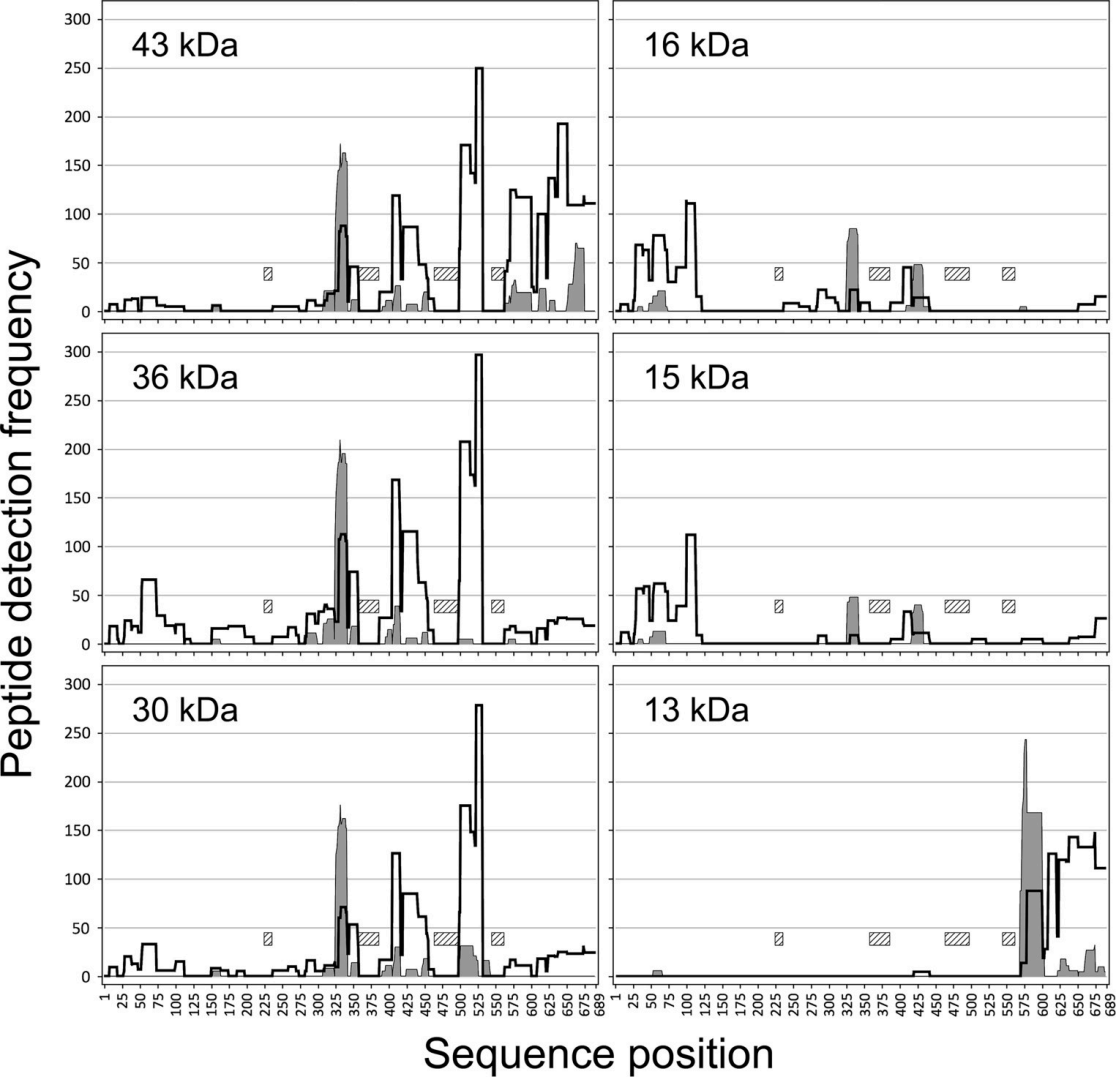
Plateau plots of peptide detection frequencies on MS. Individual panels show results obtained for the trypsinized 43, 36, 30, 16, 15, and 13 kDa bands. The white-filled plateau traces represent all peptides with N- and C-termini matching expected tryptic cleavage sites. The gray-filled plateau traces show peptides with non-tryptic N- or C-termini. Cross-hatched bars indicate the four glycosylated tryptic peptides, which were not detected due to carbohydrate-moiety heterogeneity.

Although much useful information was obtained from LC-MS runs, some ambiguity remained concerning several of the bands. In particular, each peptide map identified peptides from all regions of bLF, with MS detection frequencies ranging from very many detections to as few as one detection. A means was found to sort these signals based on the number of individual detections on the mass spectrometer, then using the multiplicity of detections to confirm or deny the presence of a given tryptic peptide in a particular fragment. The plots shown in Fig 7 display the detection frequencies as “plateaus” in which each sequence position of a given plot represents the sum of detection frequency numbers of all peptides overlapping that sequence position. Peptides matching predicted tryptic fragments are shown as white-filled plateaus, while those possessing non-tryptic N- or C-termini, presumably derived from *in vivo* nicking by VF proteases, are shown with gray fill. These plots were interpreted by assuming all signals below 50 detections were noise or cross-contamination from other fragments, while signals at 50 detections and above were confirmed to be present in that fragment.

In this group of six plots, the first, for the 43 kDa fragment, and the last, for the 13 kDa fragment, show the clearest results. In both, the tryptic-fragment profiles stay well above the 50-detection cutoff for most of their extent, while tall, gray-filled spikes mark their non-tryptic N-termini. Both start with non-tryptic N-termini and end at the authentic C-terminus of bLF. Determining their exact non-tryptic N-termini will be discussed in detail below, but major signals were seen at leucine 325 for the 43 kDa fragment and at phenylalanine 569 for the 13 kDa fragment. Their ExPASy calculated masses agree well with their PAGE-determined masses, with the 43 kDa band yielding a polypeptide weight of 39,920 plus three carbohydrates, 3 x 1,080 equals 43,160, and the 13 kDa band yielding a polypeptide weight of 13,399, with no expected carbohydrate. Additional confirming evidence comes from westerns, in which the 43 kDa fragment possesses the C-lobe epitope but not the N-lobe epitope, while the 13 kDa fragment possesses neither epitope.

Interpretation of the data for the other four fragments is complex, possibly due to cross-contamination between the bands on the PAGE gel, but substantial interpretation is still possible. In Fig 7, the 30 kDa fragment appears extremely similar to the 43 kDa fragment in its non-tryptic N-terminus, as well as in the almost identical plateaus and valleys along much of its length. However, it diverges dramatically from the 43 kDa fragment in the region of the last carbohydrate-bearing peptide, dropping to near baseline while the white-filled plateaus continue for the 43 kDa fragment. Although the missing glycopeptide confuses the issue of where the 30 kDa fragment terminates, a simple assumption gives a reasonable answer: if the glycopeptide is indeed present in the fragment, and it extends slightly farther to match the N-terminus of the 13 kDa fragment and thus ends at aspartic acid 568, its mass calculates as: polypeptide, 26,539 plus carbohydrate, 3 x 1,080, equals 29,779, a close match to its PAGE-determined mass of 30 kDa.

Results for the 36 kDa fragment were mixed. Its C-terminus could readily be interpreted as being the same as the 30 kDa fragment, because their plateau profiles are closely similar in that region. However, the N-terminus of the 36 kDa fragment presented considerable difficulty to interpret. It showed the same non-tryptic, gray-filled peak at leucine 325, but we conclude this must be cross contamination with the 44 and 31 kDa bands above and below it, because the resultant molecular weight would be identical to the 30 kDa fragment. Given that it shares its C-terminus with the 30 kDa fragment, then the extra mass can only come from the region preceding leucine 325. Although no strong white-filled or gray-filled plateaus are seen, a smaller, white-filled plateau region is indeed present on the N-terminal side of the gray-filled peak, in the 264–324 region of the plot. Compared to the 43 kDa and 30 kDa plots, this white-filled plateau feature is more substantial then in either of the other two plots. On that basis, we tentatively conclude this fragment initiates at or near tryptophan 264. If this interpretation is correct, then it would yield a calculated polypeptide molecular weight of 32,999, plus carbohydrate, 3 x 1,080, equaling 36,239, a close match to the PAGE result of 36 kDa. The data may be weak in this region if the large number of lysines and arginines yielded tryptic fragments too small to bind the C18 tip during preparation for MS. However, other choices of N-termini lead to unacceptably large mismatches between the calculated molecular weight and the PAGE results. More experimentation will be required to firmly establish the N-terminus of this fragment.

Finally, the plots for the 15 and 16 kDa bands both gave strong signals in the N-terminal region from tryptophan 8 to arginine 121. Given western reactivity of these bands with N-lobe mAb, we can confidently assign both fragments to this region of the N-lobe. However, the sequence from position 8 to 121 yields an unacceptably low calculated mass, so it is likely that the N- and/or C-termini of these fragments contain additional sequences. Two observations argue that the fragments actually start at alanine 1, the N-terminus of whole bLF: first, MS data shown below demonstrates that a number of other peptides start at alanine 1, and second, the region around their expected C-termini again has a high prevalence of lysines and arginines, so the missing MS data may be due to small tryptic peptides not bound by C18 tips. Furthermore, the expectation that the C-termini of both fragments may be non-tryptic suggests the observed C-terminal arginine 121 may not be the correct stopping point for either fragment. Given this shortcoming in the MS data, we tentatively estimated the C-termini of the 15 and 16 kDa fragments by matching ExPASy-calculated molecular weights to arbitrary sequences starting at alanine 1. These calculations yielded an approximate C-terminus at arginine 133 for the 15 kDa fragment, with a calculated molecular weight of 15,082, and a C-terminus at serine 141 for the 16 kDa fragment, with a calculated molecular weight of 16,046. These locations are only approximate, and additional experimentation will be required to unequivocally define these C-termini.

### A close-up look at non-tryptic N- and C-termini

**Fig 8:**
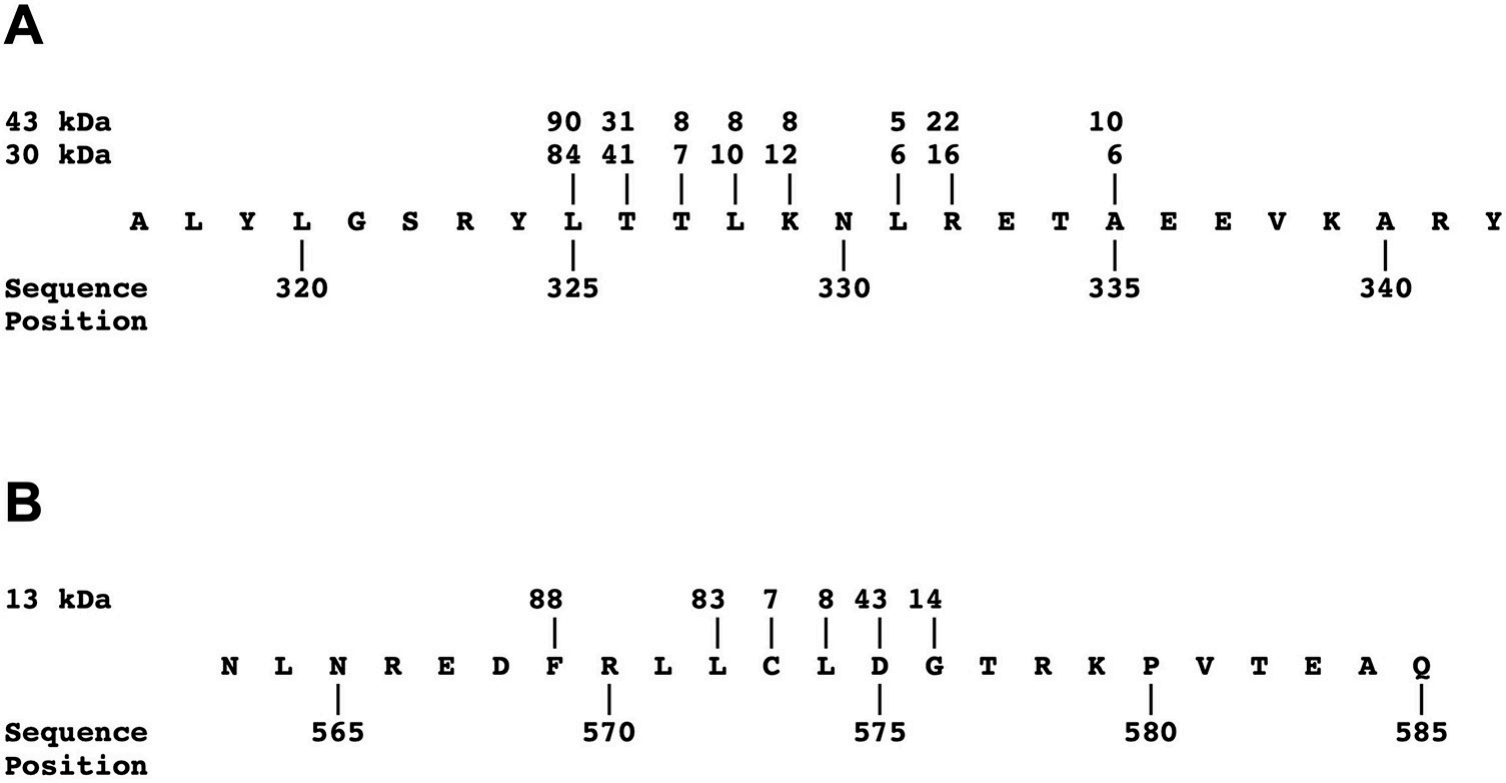
Peptides with non-tryptic N-termini. Two segments of the bLF sequence are shown, where peptides with non-tryptic N-termini were detected at high multiplicities. Panel A shows the region from sequence position 317 to 342 in which non-tryptic N-termini were found for the 43- and 30-kDa fragments, while Panel B shows the region from sequence position 563 to 585 in which non-tryptic N-termini were found for the 13-kDa fragment. Numbers above the sequences show the multiplicity of detection of peptides that start at those particular amino acids. These numbers correspond to the tallest gray-filled plateaus in the plots for these fragments in Fig 7. For clarity, positions with fewer than 5 detections are left blank.

Fig 7 showed gray-filled plateaus for the non-tryptic peptides identified by LC-MS on each fragment. Fig 8 shows the exact sequence locations of these N-termini. The largest detection numbers allow identification of N-termini at position 325 for the 43- and 30-kDa fragments (90 and 84 detections, respectively) and at position 569 for the 13 kDa fragment (88 detections). However, additional useful information can be found in other numbers shown here.

While in general most of the two regions lack detection numbers, there are clusters of N-termini detected in the segments just to the C-terminal side of the main N-termini in both fragments. We suspect these represent alternative VF endoprotease cleavage sites, or as will be seen below, the results of aminopeptidase processing of the fragment N-termini.

### Top-down mass spectrometry

To gain additional information on endo- and exopeptidase cleavages occurring in VF, we applied a different mass spectroscopic approach to the *ex vivo* digest shown in Fig 3. Top-down mass spectral analysis of the aliquots taken at 2, 4, and 24 hr identified 261 peptides, which are listed in the supplemental S1 Table. To shed light on the specificity of the proteases involved, we summed the occurrences of the 20 protein amino acids at each of the six positions before (P1-6) and after (P1-6’) the cleavage sites that generated the peptides’ N- and C-termini.

Table 1 shows the results of this analysis. While most P and P’ positions show no strong preference for particular amino acids, there were six cases where amino acids reached their peak value at either the P1 or the P1’ position. Arginine is the most prevalent amino acid at the P1 position, while the small amino acids alanine and serine dominate the P1’ position. Lysine, though not peaking at P1, is well represented, as are the large hydrophobic amino acids, phenylalanine, leucine, and tyrosine.

**Table 1:** Amino acid frequencies at N- and C-termini of fragments.

Amino Acid	P6	P5	P4	P3	P2	P1	P1’	P2’	P3’	P4’	P5’	P6’
A	34	45	33	32	54	29	107	93	37	62	34	33
C	10	11	16	7	10	14	14	9	16	7	23	8
D	37	15	20	14	59	21	17	22	47	30	30	21
E	19	44	12	17	25	15	57	16	36	10	45	47
F	16	12	12	18	18	52	25	47	12	14	60	14
G	47	46	48	27	22	17	23	16	37	46	28	64
H	41	6	2	8	28	8	7	4	34	6	0	14
I	5	15	6	6	3	3	8	3	10	4	8	8
K	58	70	29	60	20	47	12	31	31	72	83	51
L	35	50	39	54	35	50	44	37	67	69	35	44
M	5	3	4	15	2	3	1	2	2	1	2	5
N	20	3	46	11	13	13	9	30	12	11	39	17
P	22	43	16	15	8	5	10	38	12	36	10	24
Q	4	16	40	20	17	7	15	26	41	20	16	43
R	15	9	47	23	19	120	13	29	46	25	10	12
S	39	63	22	47	72	14	83	31	13	24	19	17
T	16	19	33	57	31	19	14	17	15	18	21	16
V	45	12	52	53	46	18	23	44	43	53	47	74
W	22	6	7	6	6	7	3	11	2	6	4	1
Y	8	10	14	8	11	37	37	16	9	8	7	8

Numbers highlighted in pink show where the highest occurrence of an amino acid was at the P1 position; blue indicates maximum values at the P1’ position.

**Fig 9:**
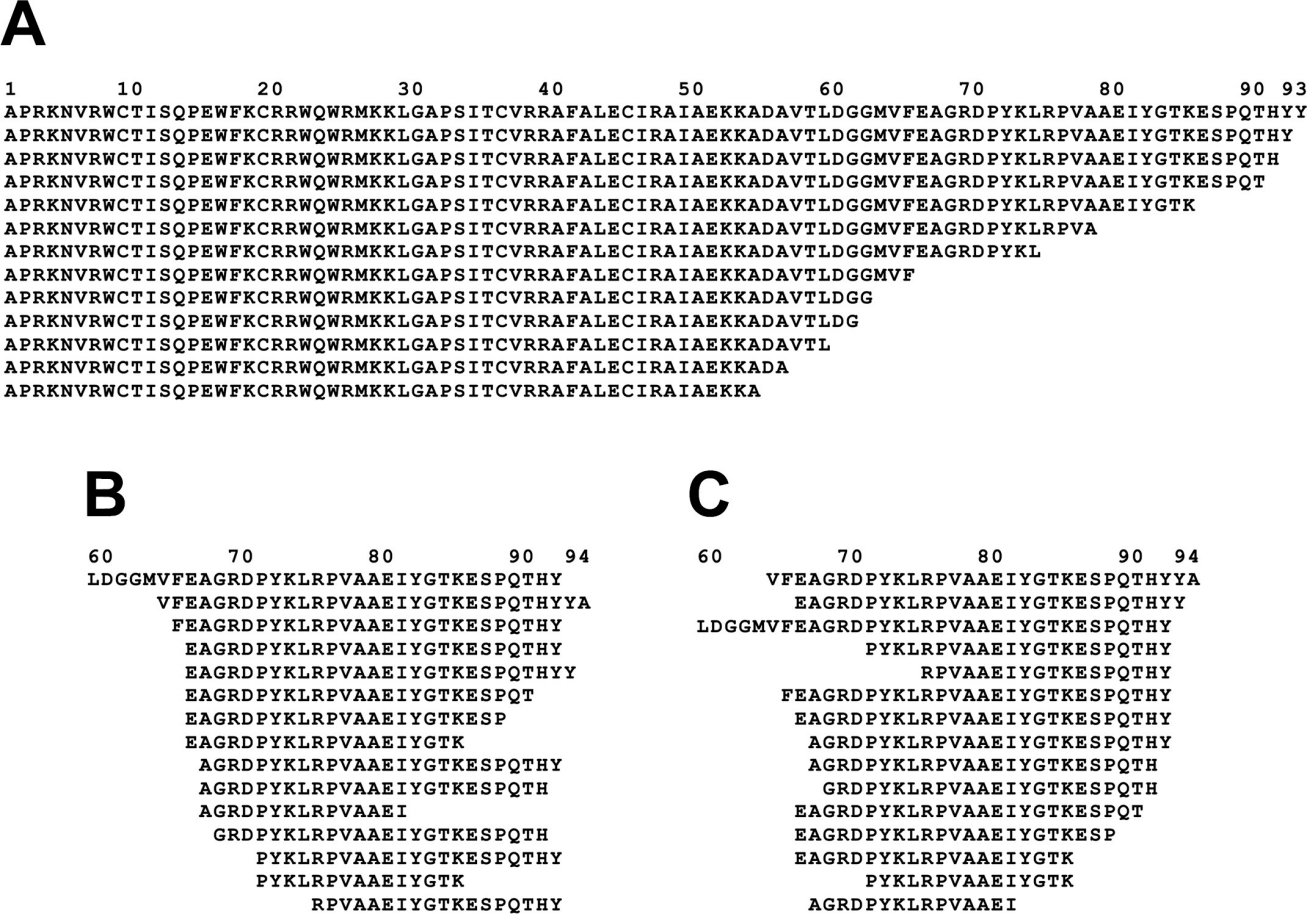
Evidence for exopeptidase processing of fragments. In panel A, a subset of fragments all starting at the N-terminus of bLF are aligned to show a series of C-terminal truncations consistent with processing of the longest fragment by carboxypeptidase(s) or other C-terminal exopeptidases. In panels B and C, fragments from the region 59–94 within bLF are arranged to show that both N-terminal truncations (B) and C-terminal truncations (C) can occur in the same set of peptides.

In addition to these putative endopeptidase cleavage sites, some peptides could be grouped into series in which N- or C-termini where truncated by removal of one or a few amino acids, suggesting the action of exopeptidases such as aminopeptidases, carboxypeptidases, and dipeptidyl exopeptidases. Fig 9 shows selected examples of these truncation series. The alignment in panel 9A shows C-terminal truncations of the N-terminal fragment of bLF. It is noteworthy that the longest sequence has a mass of 10,806, which is consistent with the prominent band seen at 11 kDa in the 24 hr lane of Fig 3. This fragment would result from a post-tyrosine endopeptidase cleavage, consistent with Table 1, to release it from bLF. Also noteworthy is that Fig 3 shows a diffuse blue-staining streak below the 11 kDa band, which might incorporate all of the shorter peptides shown in Fig 9A.

Panels B and C of Fig 9 present another group of peptides from the sequence region 59–94 of bLF. The two panels show the identical set of peptides, arranged to emphasize the N-terminal truncations (Panel B) and C-terminal truncations (Panel C), which may have been produced by aminopeptidases and carboxypeptidases trimming both ends of an endopeptidase fragment that was originally longer.

## Discussion

Degradation of bLF by vaginal fluid proteases is a complex process involving multiple endo- and exopeptidases. Nevertheless, a common set of major fragments suggests the same proteases are involved in VFs from different human subjects. The rates of proteolysis may vary between individuals, but typically a quasi-steady state develops during dose dissolution as newly dissolved bLF replaces expelled or degraded material. Consequently, both intact and proteolyzed bLF species persist for up to 24 hr post-dose, whereupon both bLF and fragments disappear concurrently with complete dissolution of the suppositories. A small percentage of the administered dose may be proteolyzed to small peptides, but the bulk is excreted either intact, nicked, or as major fragments such as the 37 kDa and 43 kDa fragments. Thus, clearance by washout predominates over proteolysis, which implies that the dosed lactoferrin retains sufficient iron-binding capacity to reduce the amount of iron available to vaginal microbes. This in turn should alter the microbiome by favoring normal, iron-independent species like lactobacilli over iron-requiring BV pathogens like *Gardnerella*.

Lactoferrin isolated from bovine milk includes a small percentage of proteolytically nicked molecules, attributed to trace amounts of proteases in milk, including plasmin, milk acid protease, and proteases derived from leukocytes [22–24]. These nicks primarily occur in the N-lobe, yielding fragments with molecular weights in the 30-to-40 kDa range for the nicked N-lobe and in the 40-to-50 kDa range for the corresponding C-lobe. The present work shows that VF proteases nick the dosed MTbLF in this same region, but at a unique site. The single major cleavage at tyrosine 324 has not been reported in any previous study of bLF proteolysis.

bLF’s two iron binding centers depend on multiple iron-ligand amino acids brought together in the 3-dimensional fold of the N- and C-lobes, and fragmentation by pepsin in stomach fluid destroys these iron centers, liberating any bound iron [1, 25, 26]. bLF fragmentation has also been investigated in biological fluids other than stomach. Rogan [27] reported cystic fibrosis lung fluid proteolysis of bLF due to Cys-cathepsins B, L, and S, as well as pseudomonas proteases and human neutrophil elastase. Dashper [28] reported bLF fragmentation by proteases of the gingivitis organism *Porphyromonas gingivalis*. *In vitro* studies of vaginally pathogenic *Candida* strains showed that aspartyl proteases of the SAP family can cleave lactoferrin as well [28]. Investigation of bLF fragmentation *in vitro* by trypsin demonstrated major fragments of approximately 30 and 50 kDa, which remained associated under mild conditions, and which retained their iron-binding ability [29]. More extensive trypsinization produced smaller fragments with molecular weights of 21, 38, and 45 kDa [25]. The iron binding status of these latter fragments is unknown.

### Proteolysis model for bovine lactoferrin in vaginal fluid

**Fig 10:**
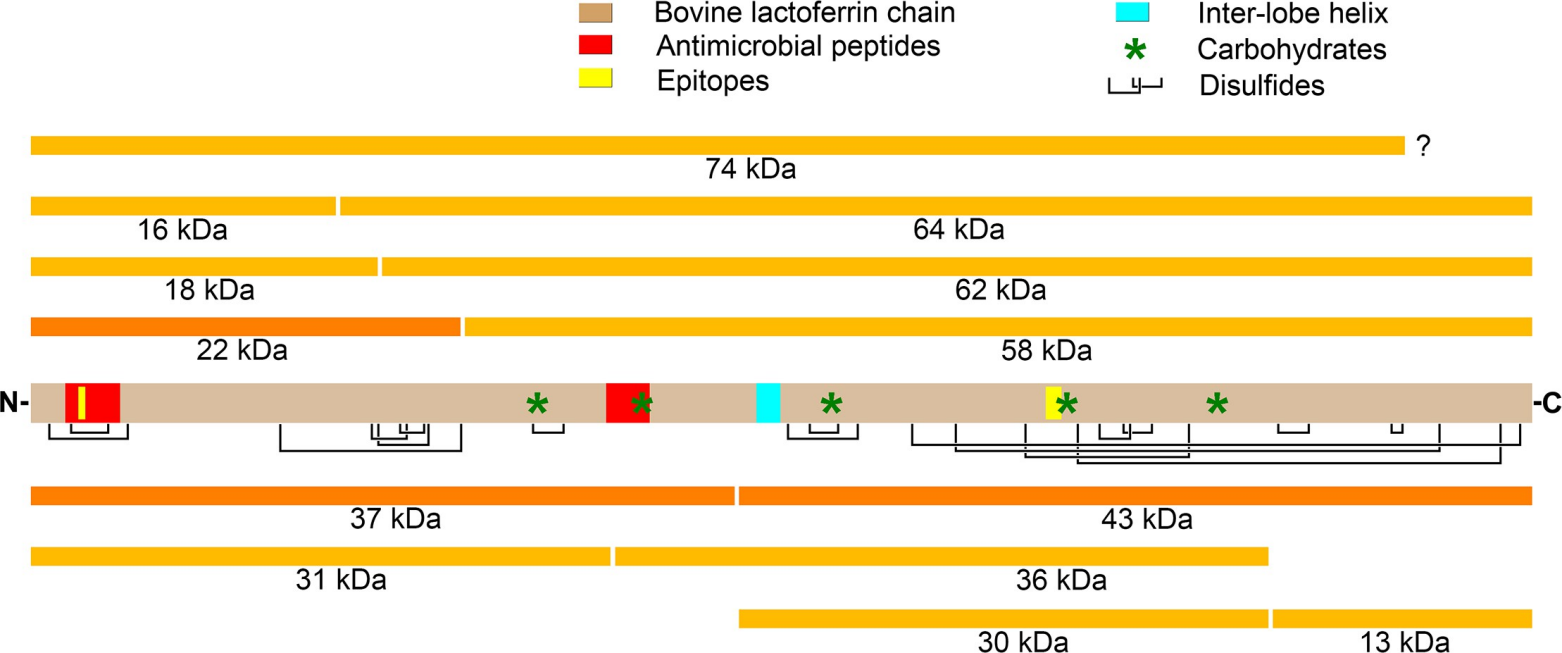
Fragmentation model for bLF in vaginal fluid. The central gray bar represents whole lactoferrin showing disulfide bonds (black lines), carbohydrate moieties (green asterisks), epitopes (yellow), and the antimicrobial peptides lactoferricin B (left) and lactoferrampin (middle) in red. The N- and/or C-termini of some fragments are approximate, their positions estimated only from PAGE and Western gels, which are only accurate to plus-or-minus 1–2 kDa. Others were defined precisely by mass spectroscopy as described in the text.

Fig 10 presents a model of the major fragments of bLF produced in VF. The most prevalent fragments are indicated by dark orange color while fragments present in lesser amounts are shown in light orange.

A number of conclusions can be drawn from our proteolysis data. These include:

#### An X + Y = 80 kDa relationship is commonly observed among fragments

As noted in the experimental results and depicted graphically in Fig 10, there are several examples where two fragments can be explained as the N- and C-terminal pieces of bLF cleaved at a single location by an endopeptidase. The most prominent example is the N-terminal 37 kDa fragment matched with the 43 kDa C-terminal fragment. These clearly fit the pattern, X + Y = 80, and tend to be prevalent on most, but not all PAGE and western gels. These two fragments are particularly interesting in that they represent intact, or nearly intact, N- or C-lobes of the molecule, and each possesses the full set of iron ligand amino acids. Both fragments are of the correct ~40 kDa weight to have been responsible for the second iron-binding peak in the SEC HPLC runs in Fig 4.

Other pairs totaling 80 kDa were also seen. For example, the prominent bands at 16 and 18 kDa match a prominent doublet band at 62 and 64 kDa, to yield 16 + 64 = 80 and 18 + 62 = 80. Also, the often prominent 22 kDa band is sometimes matched with a 58 kDa band, again yielding 22 + 58 = 80. However, some prominent bands do not correspond to this X + Y = 80 pattern. For instance, the 31 kDa N-lobe fragment often does not have a counterpart at 49 kDa. This is not problematic if the 31 kDa segment remains intact while its 49 kDa partner is further cleaved into sub-fragments. Similarly, the 22 kDa fragment sometimes exists in the absence of substantial 58 kDa fragment, presumably due to degradation of the 58 kDa fragment while the 22 kDa fragment remains intact.

In addition to the above-mentioned pairs, which each have one epitope from either the N- or C-lobe, there is one other substantial fragment that possesses both epitopes, namely the 74 kDa band seen just below intact 80 kDa bLF in the reduced lane of Fig 2. This implies a corresponding 6 kDa fragment to yield 6 + 74 = 80 kDa, and indeed, a 6 kDa band is seen on that gel and some others. Furthermore, it is possible to assign the cleavage responsible for this fragment to a region very near the C-terminus of bLF, based on the following logic: if 6 kDa were removed from the N-terminus of bLF, then the N-lobe epitope would be excised along with it. But this is not the case and, as mentioned, the 74 kDa band stains for both epitopes. Therefore the 6 kDa portion must be cut from the C-terminal end of the molecule, yielding the 74 kDa double-epitope fragment plus a non-epitope band at 6 kDa. A candidate Coomassie blue band can be seen in Figs 2, 5 and 6.

#### Further nicking and exopeptidase trimming occur within bLF fragments

The 43 kDa band in Fig 2 is prominent when non-reduced on the left, but nearly vanishes when reduced on the right. We interpret this to mean that reduction of disulfides releases many smaller sub-fragments, most of which do not possess the C-lobe epitope in the right-hand lane. The exopeptidase trimming demonstrated in Fig 9 would happen subsequent to additional endopeptidase cleavages. Conceivably, such trimming could occur either on free individual peptides, or on the disulfide-linked nicked forms or nicked 80 kDa bLF itself. Evidence of this is seen in streaks of presumably trimmed material below the main bands in Figs 1–3, especially in late digestion time points.

#### Antimicrobial peptides lactoferricin B and lactoferrampin were not detected

Although proteolysis can destroy bLF’s iron-binding capacity, several sub-fragments possess antimicrobial activity due to short, polycationic sequences that bind directly to microbial cell surfaces [30–35]. Bellamy and coworkers used pepsin hydrolysis to generate lactoferricin B, a small fragment of the N-lobe with bactericidal activity 10-fold greater than native 80 kDa bLF. Another antimicrobial peptide, lactoferrampin, was derived from of bLF’s N-lobe by chemical synthesis of a poly-basic region [36].

We did not identify either of these peptides in our experiments, suggesting that these antimicrobial substances do not play a significant role in microbiome alteration of VF by MTbLF. While western blots in Figs 2 and 5 were clearly able to identify a variety of lactoferricin B-containing fragments, neither show staining in the 3.1 kDa region at the bottom of the gels. We could easily detect lactoferricin B in peptic digests under similar loading and staining conditions. Therefore, we conclude that no significant amounts of lactoferricin B are produced by VF proteases. No evidence of lactoferrampin [36] was detected either, although with no mAb to identify it, its presence or absence in VF digests of bLF is less certain.

#### Polypeptide sequences and a three-dimensional model of proteolysis

**Fig 11:**
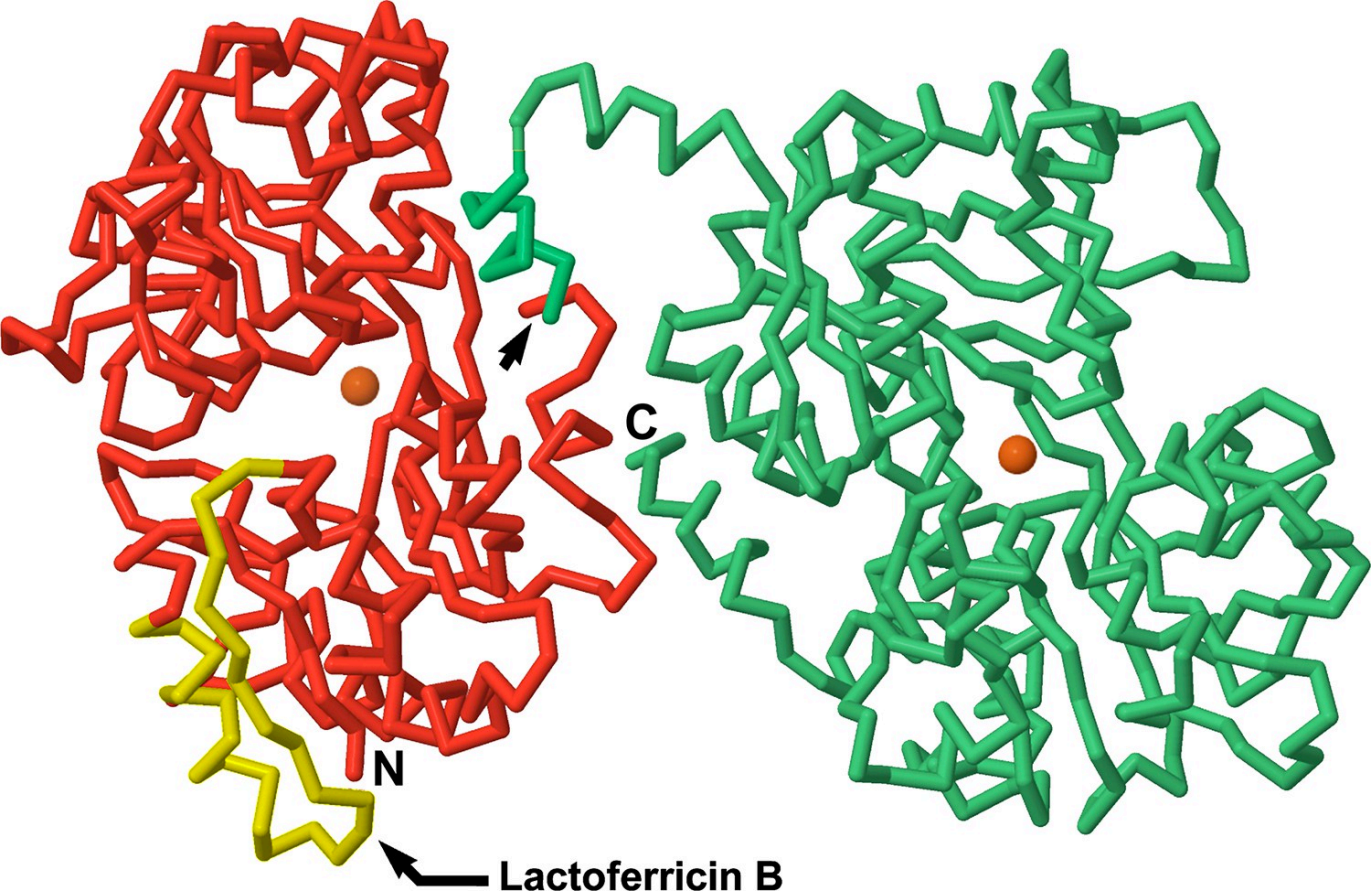
Three-dimensional image of the largest fragments. The 37 kDa N-lobe fragment is shown in red-and-yellow, and the 43 kDa C-lobe fragment is shown in green. The arrow near the center of the image shows the single peptide bond that was clipped to generate the two fragments. The iron atoms bound within each lobe are shown as orange spheres. The antimicrobial peptide portion of the N-lobe, lactoferricin B, is highlighted in yellow. It remains an integral portion of the 37 kDa fragment and reacts with its mAb on western blots. The C-lobe antigenic site is integral to its fragment as well. It is located on the far side of the green polypeptide segment and is not visible here.

Fig 11 shows a 3D image of bLF with the two major fragments indicated in red for the 37 kDa N-lobe fragment and green for the 43 kDa C-lobe fragment. The cleavage does not clip the protein into two equal 40 kDa halves, but slightly off-center and within the N-lobe. However, both fragments retain their full set of four iron-ligand amino acids, allowing them to retain the iron binding demonstrated on SEC HPLC in Fig 4. The 3D image makes it clear that the single peptide bond cleavage that generates these two fragments separates them into two essentially intact iron binding domains, and that multiple strands of polypeptide intervene between the clip site and the iron binding sites.

**Fig 12:**
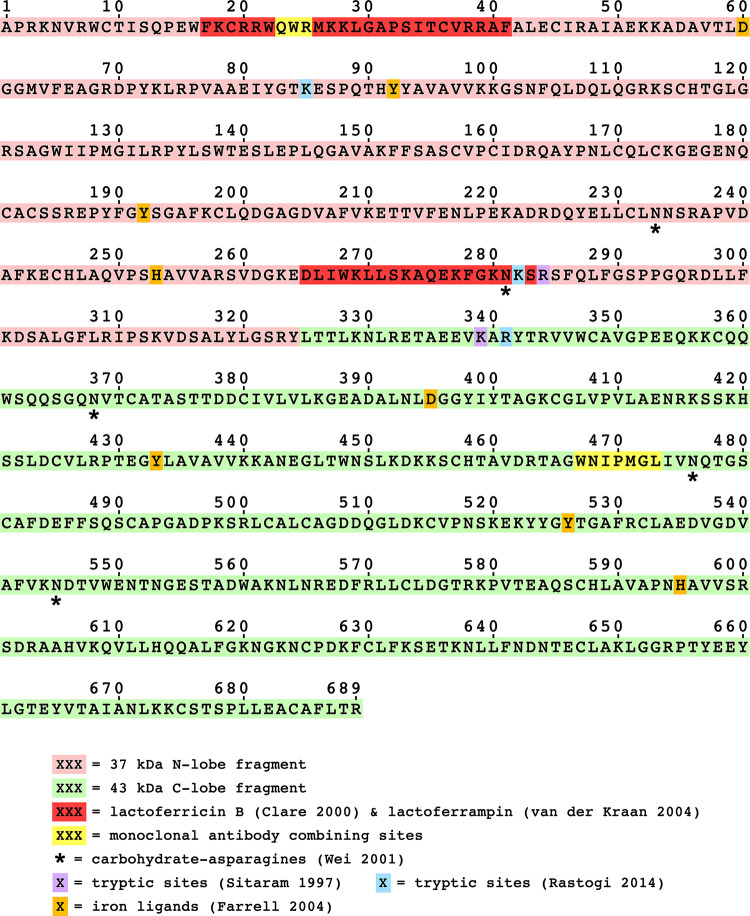
Sequence features of bLF and its VF fragments. The 37 kDa N-lobe fragment is highlighted in pink and the 43 kDa C-lobe fragment in green. Various other structural features are mapped onto the sequence of bLF as noted in the legend: epitopes are highlighted in yellow, antimicrobial peptides are shown in red (lactoferricin B above, lactoferrampin below), carbohydrate-bearing asparagine (N) amino acids are noted with asterisks, basic amino acids targeted by limited tryptic digests are noted in purple [37] or blue [25]. The two identical homologous sets of iron-ligand amino acids are highlighted in orange; four occur in the 37 kDa fragment and four in the 43 kDa fragment.

Fig 12 highlights the individual iron ligand amino acids within the two lobes, along with the locations of epitopes, carbohydrate moieties, and other molecular features. Notable are several basic amino acids, lysine (K) and arginine (R), that have been reported to produce large fragments of bLF by trypsin [26, 33, 37]. Clearly, the enzyme(s) responsible for vaginal bLF proteolysis have different target specificities. Correspondingly, the major cleavage at tyrosine 324 in the present work lies a substantial distance from either of the reported trypsin attack sites following lysine 282 / arginine 284 or lysine 339 / arginine 341. Also notable is that trypsin also cleaves at lysine 85 [26] yielding a 21 kDa N-terminal fragment similar to our 22 kDa fragment, both of which contain the N-lobe mAb binding site. Although we do not have a specific identity for this VF cleavage site, it is unlikely to match lysine 85 because of its heavier 22 kDa size. It is worth noting that cleavage in this region by either trypsin or VF proteases seems likely to disrupt N-lobe iron binding by separating one of the four iron ligands, aspartic acid 60, from the others.

#### Potential identities of the responsible proteases

Finally, it is worth considering the identities of the VF proteases responsible for the fragmentation patterns we have observed. Recent proteomics analysis of VF demonstrated the presence of many proteases, especially serine- and cysteine-class enzymes as well as a large number of kallikrein family members, although most of these should be deactivated at low pH [38, 39]. In contrast, the seminal aspartyl proteinase, gastricsin, can be activated by the low pH of VF and remain active for hours [40].

Table 1 shows both basic amino acids and large hydrophobics at P1, suggesting trypsin- and chymotrypsin-like activities, respectively. However, this *ex vivo* digest was carried out at pH 3.5, where both enzymes would be inactivated by acid. Therefore, alternative enzymes are likely. For example, cathepsins D and E, members of the pepsin family of aspartyl proteases, have pH-activity profiles compatible with these results, and substrate specificities that include post-leucine, post-phenylalanine, and pre-tyrosine [41, 42]. Several members of the cys-cathepsin family of proteases are active at acid pH and can cleave after arginines or lysines [43, 44]. However, the poor correlation of aliphatic amino acids leucine, isoleucine and valine at position P2 makes cys-cathepsin involvement less likely [45]. The occurrence of aromatic amino acids phenylalanine and tyrosine at P1 suggests the specificity of chymase or cathepsin G [46] although again, low pH would inhibit these serine proteases. Transmembrane serine proteases attached to vaginal epithelial cells have been reported, which possess strong preferences for arginine [47], but these would also be inhibited at low pH. The seminal acid protease, gastricsin [40], shares specificities with cathepsins D and E. Amino- and carboxy-peptidases are implicated as well, although the specific enzymes cannot yet be identified. Further experimentation will be needed to clarify the exact nature and number of proteases involved.

#### Tricolor western blots

The tricolor western technique, developed during these studies, may find wider applicability in protein metabolism investigations. Researchers often study recombinant proteins with antisera to multiple epitopes, or with N-terminal and/or C-terminal epitope tags attached. This new method represents a convenient way to visualize such double-labeled proteins and follow their proteolysis or other reactions in cells, tissues, or biological fluids.

## Conclusions

This report describes the fragmentation patterns seen when bLF is dosed vaginally in clinical subjects or incubated *ex vivo* with vaginal fluid. A consensus pattern of polypeptide metabolites was observed. The 80 kDa bLF molecule is initially cleaved between its N- and C-lobes, then degraded into sub-fragments and ultimately to small peptides. We found that most VF fragmentation patterns included large amounts of an N-lobe 37 kDa fragment and a C-lobe 43 kDa fragment resulting from a single cleavage following tyrosine 324. These large fragments possessed full sets of iron-ligand amino acids and retained their iron-binding ability. In some VF samples, alternative forms of large fragments were found, which like the 37 + 43 pair, also totaled to 80 kDa, including 58 + 22, 18 + 62, and 16 + 64 kDa forms. In general, the smaller component was from the N-lobe and the larger from the C-lobe. The 18 + 62 pair was absent in some VF samples but highly abundant in others. This variability implies that more than one endoprotease is involved, and the 18 kDa fragment’s presence depends on the balance of enzymes. Further action of VF proteases produced smaller peptide fragments from the larger ones, and we found evidence of exopeptidases trimming the N- and C-termini of these sub-fragments, reaffirming that the breakdown of bLF by VF is a multi-enzyme process. While the identities of the VF proteases responsible will require additional experimentation to elucidate, the present experiments advance our understanding of the process considerably. In addition, the tricolor western technique may be broadly applicable to protein metabolism studies, especially those in which both N- and C- terminal epitope tags are used.

## Supporting information

S1 Fig Original images for PAGE gels and western blots used in Figs 1–3, 5, 6 and supplement S2 Fig. (PDF)

S2 Fig Method for creating tricolor PAGE/western images. In panel A, selected lanes from PAGE Au26 (lanes 5–9, Coomassie blue stained) western blot Au38 (lanes 8–12, N-lobe mAb stained) and western blot Au37 (lanes 8–12, C-lobe mAb stained) are shown after being converted to black-and-white in Photoshop. In panel B, the images were inverted to black backgrounds and white-toned bands. In panel C, the images were converted to negative monochrome colors using Photoshop’s photo filter. In panel D, the three monochrome images were superimposed (left) and then inverted for the final composite tricolor image (right). Details of this procedure are given in the Methods section.
(TIF)

S1 Table Summations of amino acids at N- and C-termini of bLF fragments. Twenty sheets named for amino acids contain two listings of P’ + P data, one for the N-terminus of each mass spectrometry-determined peptide and one for the C-terminus of that peptide. The 261 individual peptides identified in the 2, 4, and 24 hr VF digests are listed on individual rows. Their N- and C-terminal sequence positions are listed in Column I and Column X, respectively. Excel then extracted the 6 amino acids preceding and following the cut site and placed them in adjacent columns, H and J, and W and Y. Excel then polled these hexapeptides at each position and entered a 1 in the corresponding P1-P6 or P1’-P6’ columns where there was a match to the amino acid named on that sheet. Cells not changed to 1 remained at 0. After all columns were specified as 1 or 0, the columns were summed to give the frequency numbers at the bottom. These summed frequencies were gathered into the “aa Sums” sheet, which represents the total times each amino acid occurred at each P or P’ position, for use in Table 1.
(XLSX)
