# Supplementary material for: Characterization of proteolytic degradation products of vaginally administered bovine lactoferrin
Source: PLoS One. 2022 May 19;17(5):e0268537. doi: 10.1371/journal.pone.0268537 (PMC9119511; doi:10.1371/journal.pone.0268537)
Supplement: S1 Fig — (PDF) [file pone.0268537.s001.pdf]

PAGE Au10 used in Figure 2

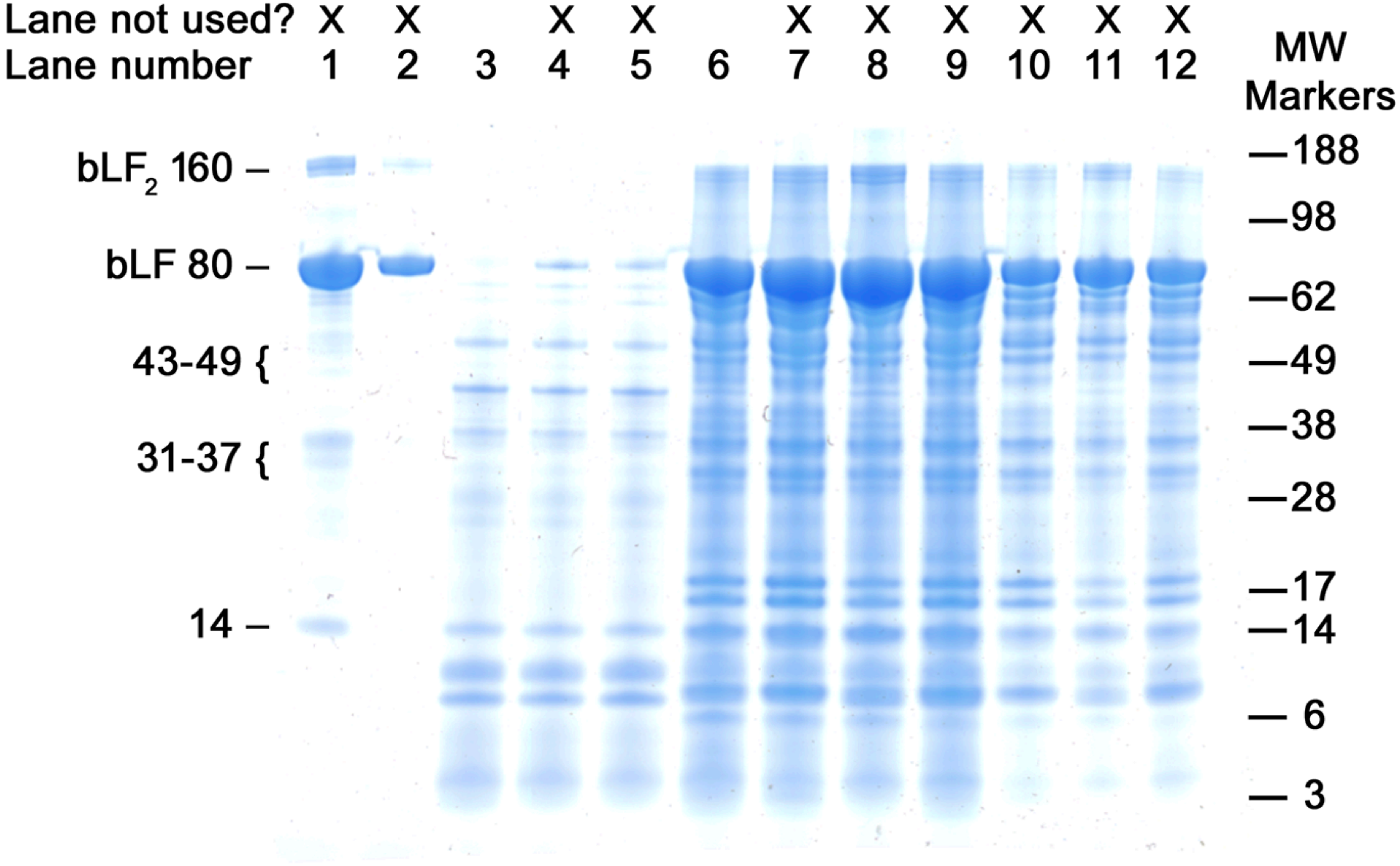

PAGE Au10 Subject 7 VF Samples		
**Au10 Lane**	**Sample**	**Fig 2 Lane**
1	MTbLF 5 µg	—
2	MTbLF 1 µg	—
3	VF 0 hr	1
4	VF 2 hr	—
5	VF 4 hr	—
6	VF 12 hr	3
7	VF 18 hr	—
8	VF 30 hr	—
9	VF 48 hr	—
10	VF 18 hr (1:4)	—
11	VF 30 hr (1:4)	—
12	VF 48 hr (1:4)	—

Notes: Coomassie Blue stained PAGE gel. bLF standard molecular weights shown at left. SeeBlue standard weights at right. Table lists sample identities and lane order in Figure 2.

PAGE Au15 used in Figure 1

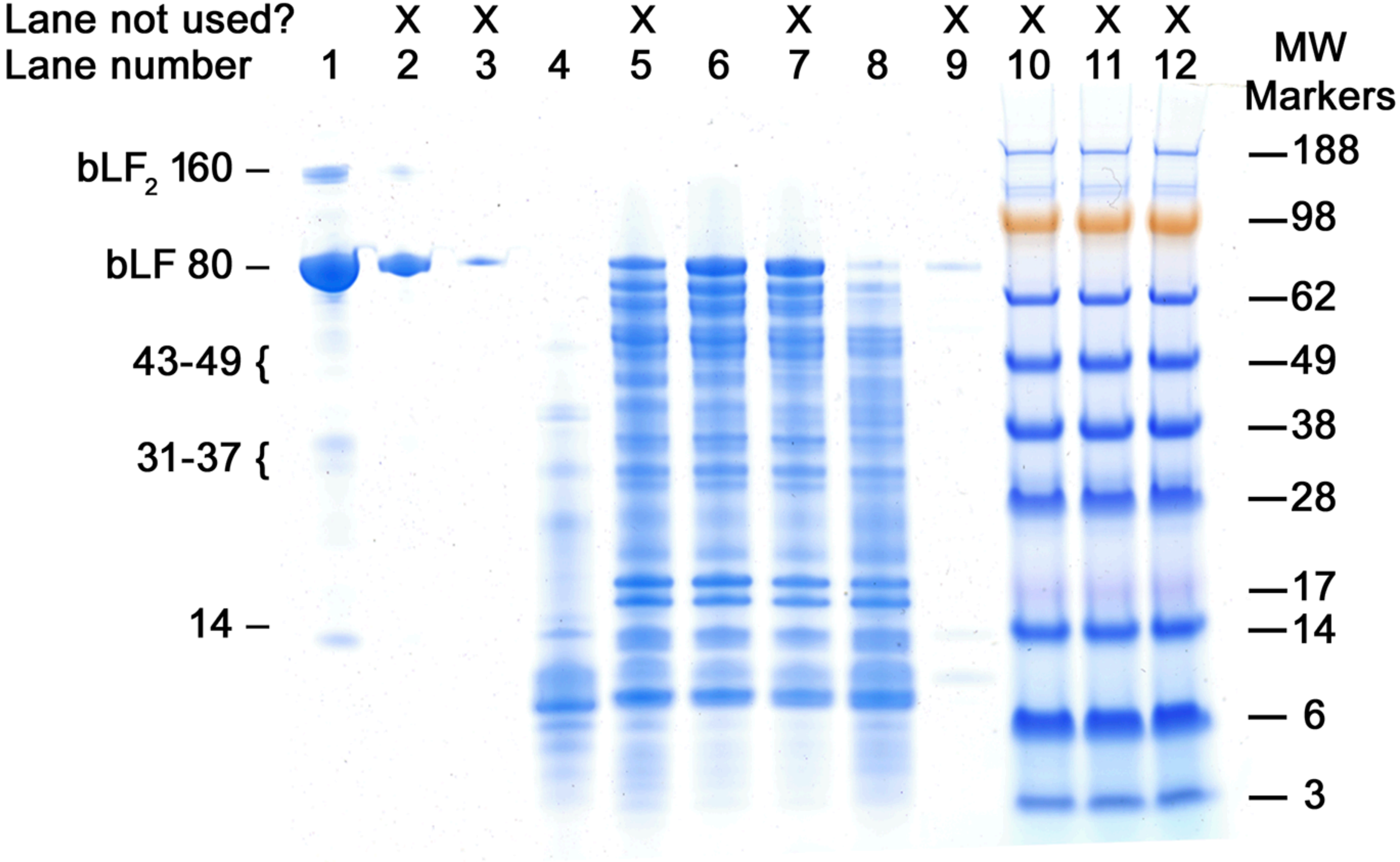

PAGE Au15 Subject 4 VF Samples		
Au15 Lane	Sample	Fig 1 Lane
1	MTbLF 5 µg	6
2	MTbLF 1 µg	—
3	MTbLF 0.1 µg	—
4	VF 2 hr	5
5	VF 8 hr	—
6	VF 12 hr	3
7	VF 18 hr	—
8	VF 24 hr	4
9	VF 30 hr	—
10	SeeBlue+2	—
11	SeeBlue+2	—
12	SeeBlue+2	—

Notes: Coomassie Blue stained PAGE gel. bLF standard molecular weights shown at left. SeeBlue standard weights at right. Table lists sample identities and lane order in Figure 1.

PAGE Au16 used in Figure 1

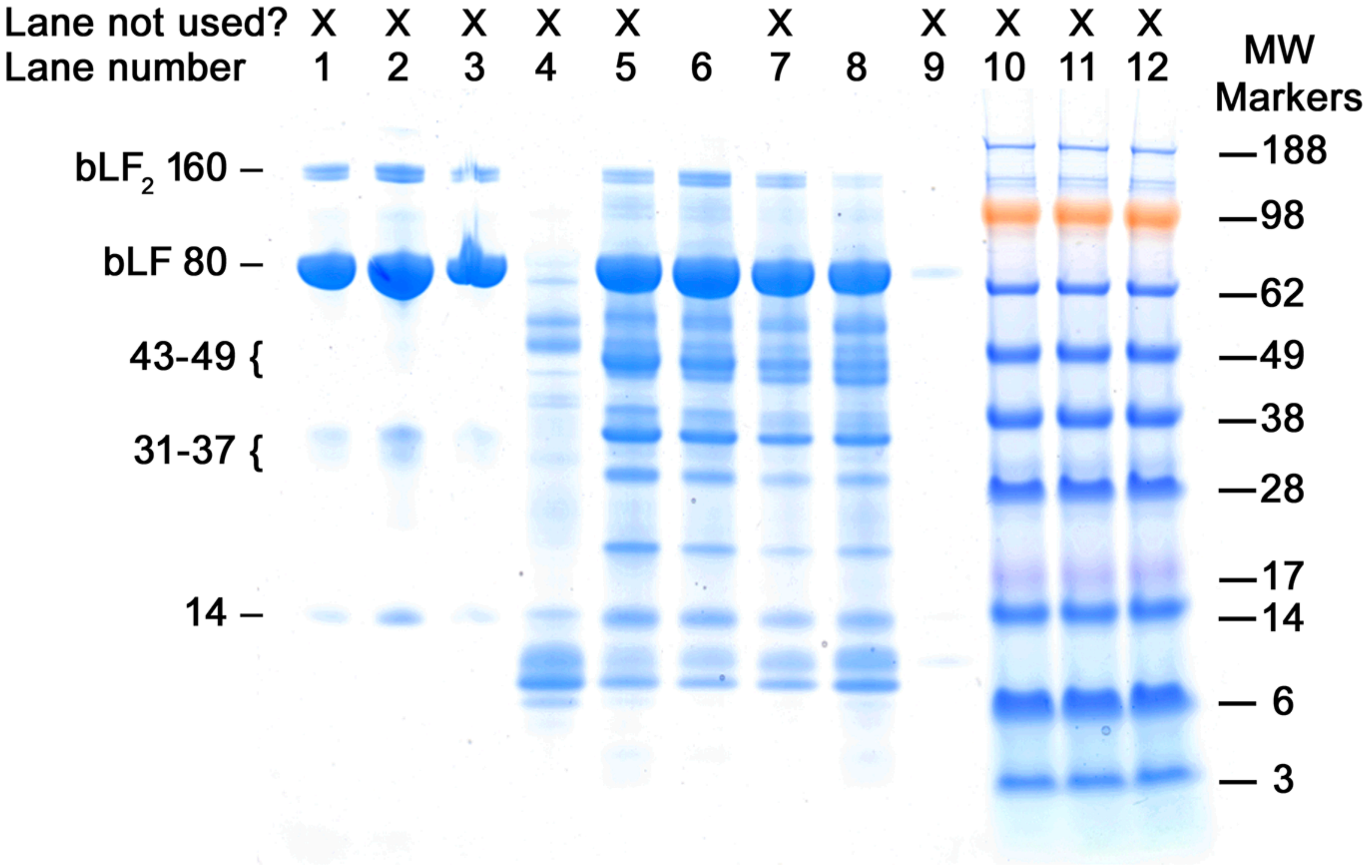

PAGE Au16 Subject 4 VF Samples		
**Au16 Lane**	**Sample**	**Fig 1 Lane**
1	MTbLF 5 μg	—
2	MTbLF 1 μg	—
3	MTbLF 0.1 μg	—
4	VF 2 hr	—
5	VF 8 hr	—
6	VF 12 hr	1
7	VF 18 hr	—
8	VF 24 hr	2
9	VF 30 hr	—
10	SeeBlue+2	—
11	SeeBlue+2	—
12	SeeBlue+2	—

Notes: Coomassie Blue stained PAGE gel. bLF standard molecular weights shown at left. SeeBlue standard weights at right. Table lists sample identities and lane order in Figure 1.

PAGE Au26 used in Figures 2 and S2

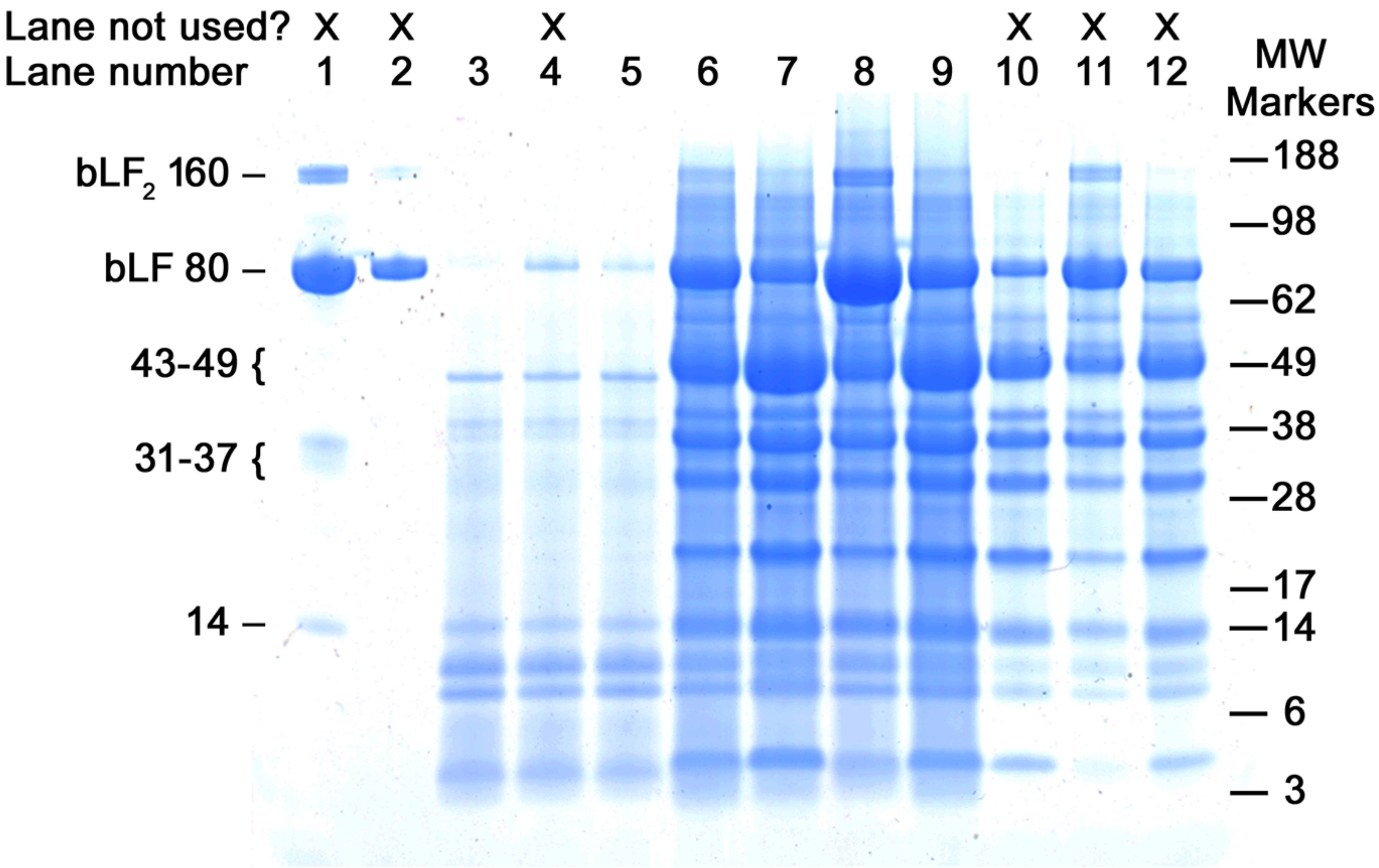

PAGE Au26 Subject 7 VF Samples			
Au26 Lane	Sample	Fig 2 Lane	Fig S2 Lane
1	MTbLF 5 μg	—	—
2	MTbLF 1 μg	—	—
3	VF 0 hr	1	—
4	VF 2 hr	—	—
5	VF 4 hr	—	1
6	VF 12 hr	2	2
7	VF 18 hr	—	3
8	VF 30 hr	—	4
9	VF 48 hr	—	5
10	VF 18 hr (1:4)	—	—
11	VF 30 hr (1:4)	—	—
12	VF 48 hr (1:4)	—	—

Notes: Coomassie Blue stained PAGE gel. bLF standard molecular weights shown at left. SeeBlue standard weights at right. Table lists sample identities and lane order in Figures 2 and S2.

Western Au37 used in Figures 2 and S2

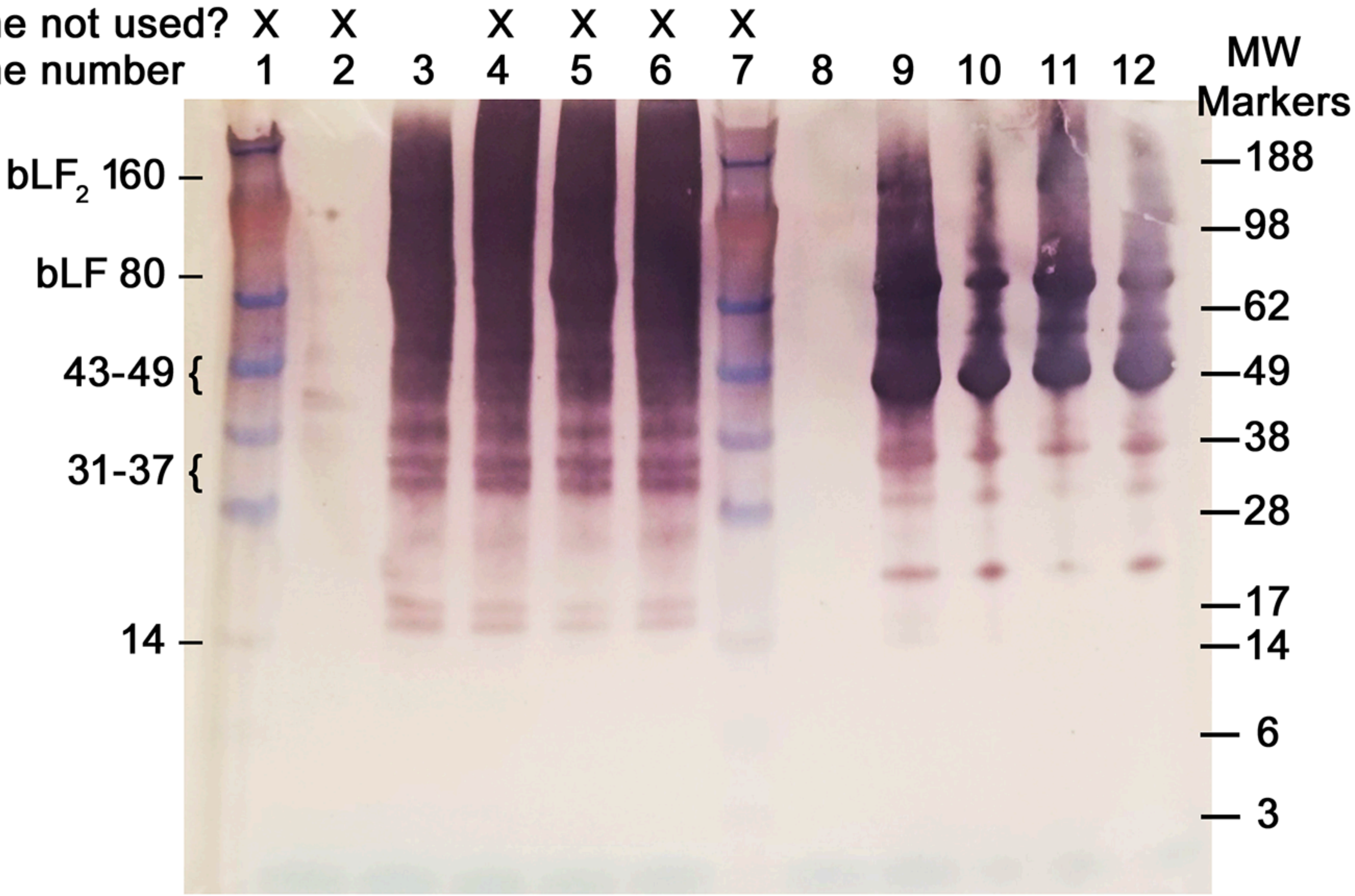

Western Au37 Subject 7 VF Samples			
Au37 Lane	Sample	Fig 2 Lane	Fig S2 Lane
1	SeeBlue+2	—	—
2	VF 0 hr reduced	—	—
3	VF 12 hr reduced	3	—
4	VF 18 hr reduced	—	—
5	VF 30 hr reduced	—	—
6	VF 48 hr reduced	—	—
7	SeeBlue+2	—	—
8	VF 0 hr non-red.	1	1
9	VF 12 hr non-red.	2	2
10	VF 18 hr non-red.	—	3
11	VF 30 hr non-red.	—	4
12	VF 48 hr non-red.	—	5

Notes: Western blot developed with anti-C lobe mAb. bLF standard molecular weights shown at left. SeeBlue standard weights at right. Table lists sample identities and lane order in Figures 2 and S2.

Western Au38 used in Figures 2 and S2

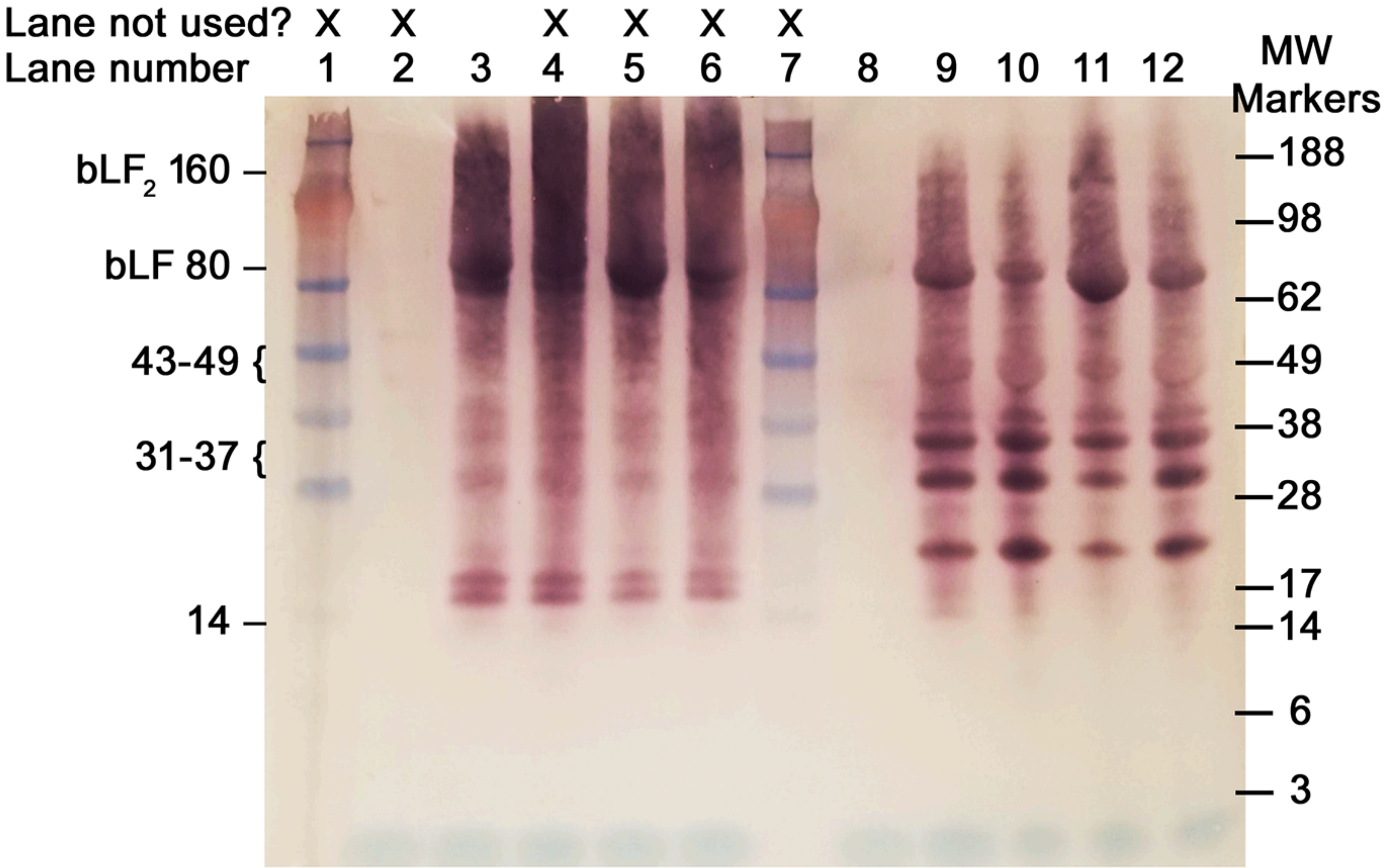

Western Au38 Subject 7 VF Samples			
Au38 Lane	Sample	Fig 2 Lane	Fig S2 Lane
1	SeeBlue+2	—	—
2	VF 0 hr reduced	—	—
3	VF 12 hr reduced	3	—
4	VF 18 hr reduced	—	—
5	VF 30 hr reduced	—	—
6	VF 48 hr reduced	—	—
7	SeeBlue+2	—	—
8	VF 0 hr non-red.	1	1
9	VF 12 hr non-red.	2	2
10	VF 18 hr non-red.	—	3
11	VF 30 hr non-red.	—	4
12	VF 48 hr non-red.	—	5

Notes: Western blot developed with anti-N lobe mAb. bLF standard molecular weights shown at left. SeeBlue standard weights at right. Table lists sample identities and lane order in Figures 2 and S2.

PAGE L47 used in Figure 3

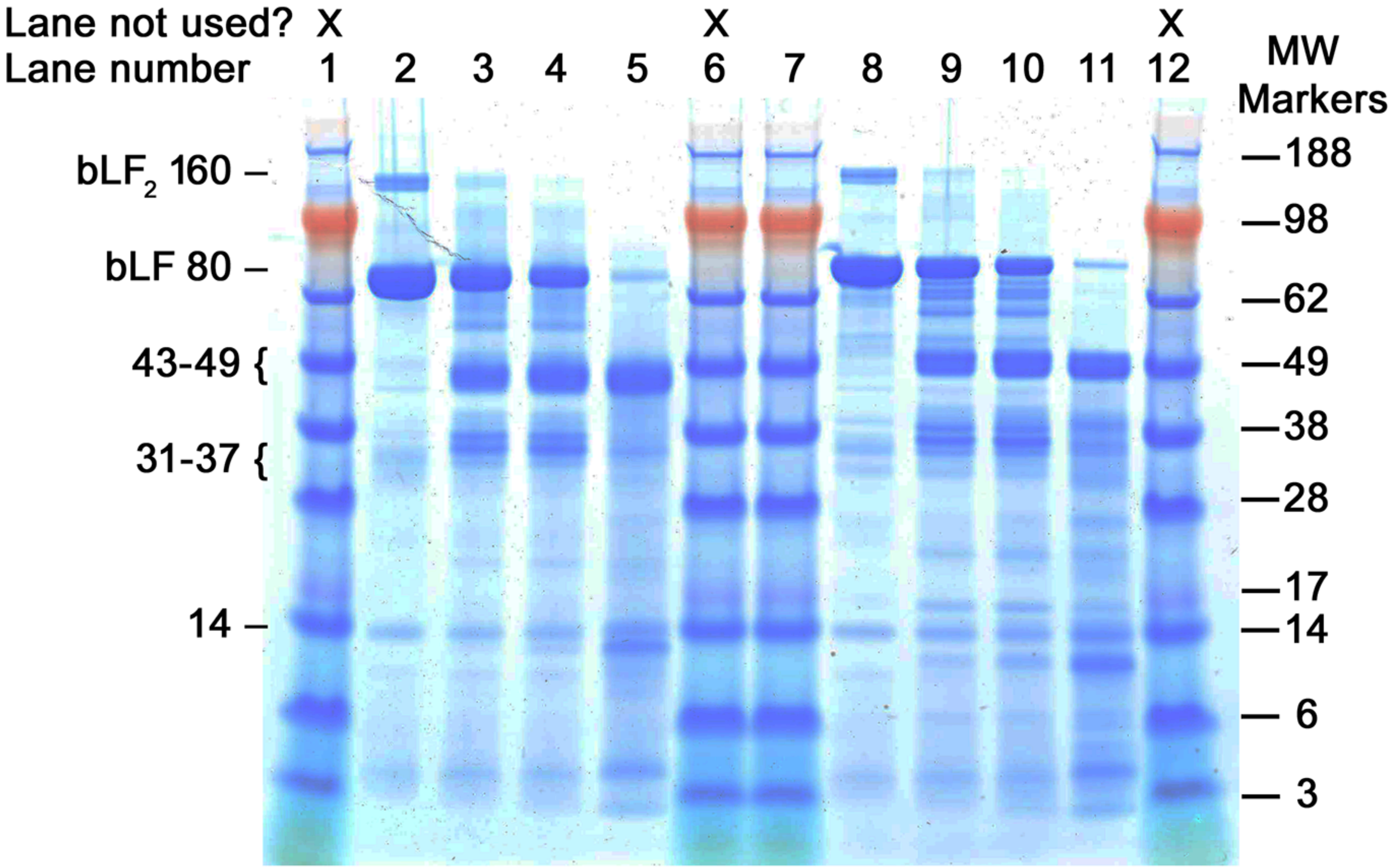

PAGE L47 *Ex Vivo* Digest Samples		
L47 Lane	Digest Time Point	Fig 3 Lane
1	SeeBlue+2	—
2	0 hr non-reduced	1
3	2 hr non-reduced	2
4	4 hr non-reduced	3
5	24 hr non-reduced	4
6	SeeBlue+2	—
7	SeeBlue+2	5
8	0 hr reduced	6
9	2 hr reduced	7
10	4 hr reduced	8
11	24 hr reduced	9
12	SeeBlue+2	—

Notes: Coomassie Blue stained PAGE gel. bLF standard molecular weights shown at left. SeeBlue standard weights at right. Table lists sample identities and lane order in Figure 3.

PAGE L51 used in Figure 5B

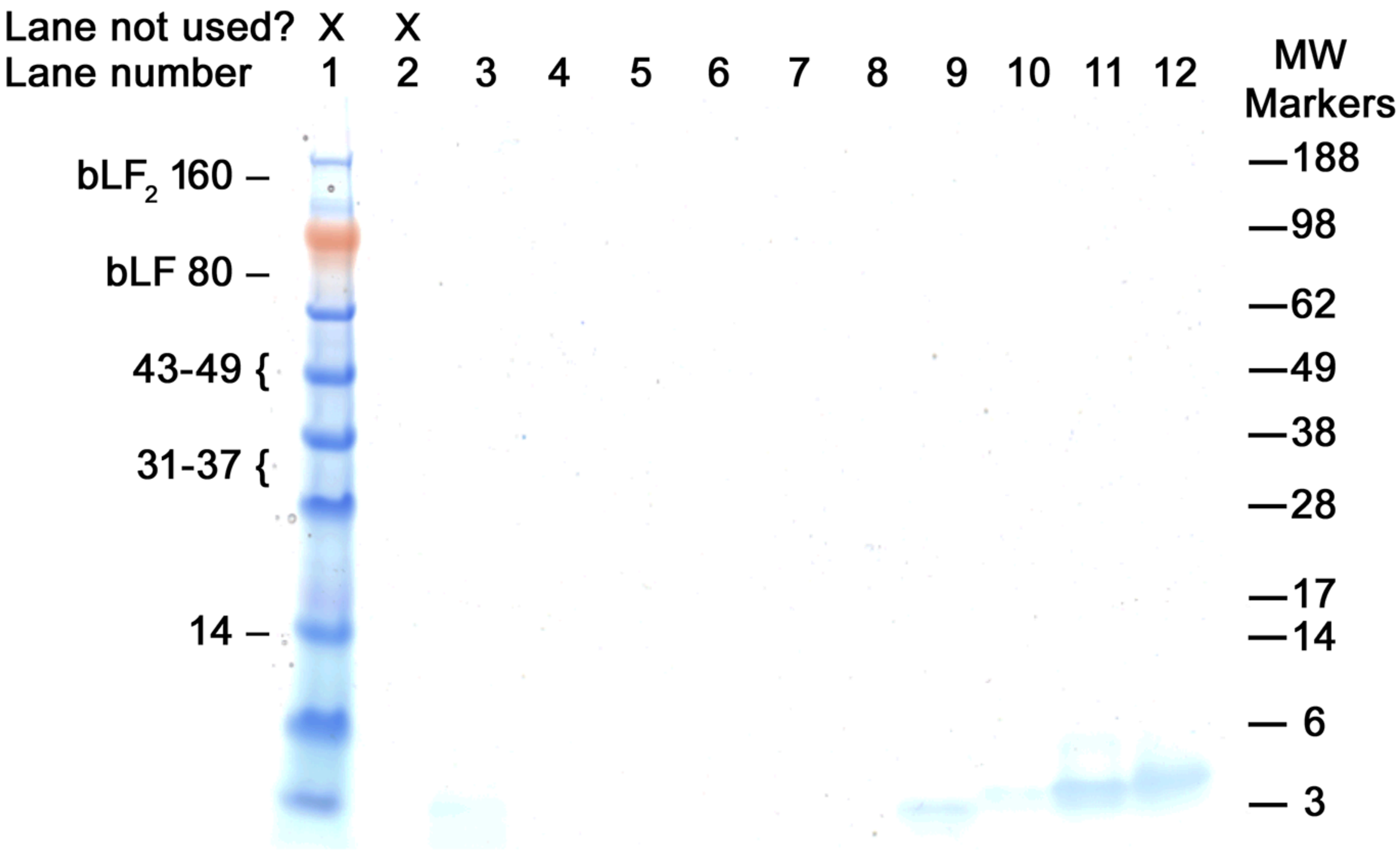

PAGE L51 HPLC Fractions		
L51 Lane	Fraction Number	Fig 5B Lane
1	SeeBlue+2	—
2	3	—
3	4	1
4	5	2
5	6	3
6	7	4
7	8	5
8	9	6
9	10	7
10	11	8
11	12	9
12	13	10

Notes: Coomassie Blue stained PAGE gel. bLF standard molecular weights shown at left. SeeBlue standard weights at right. Table lists sample identities and lane order in Figure 5B.

PAGE L52 used in Figure 5B

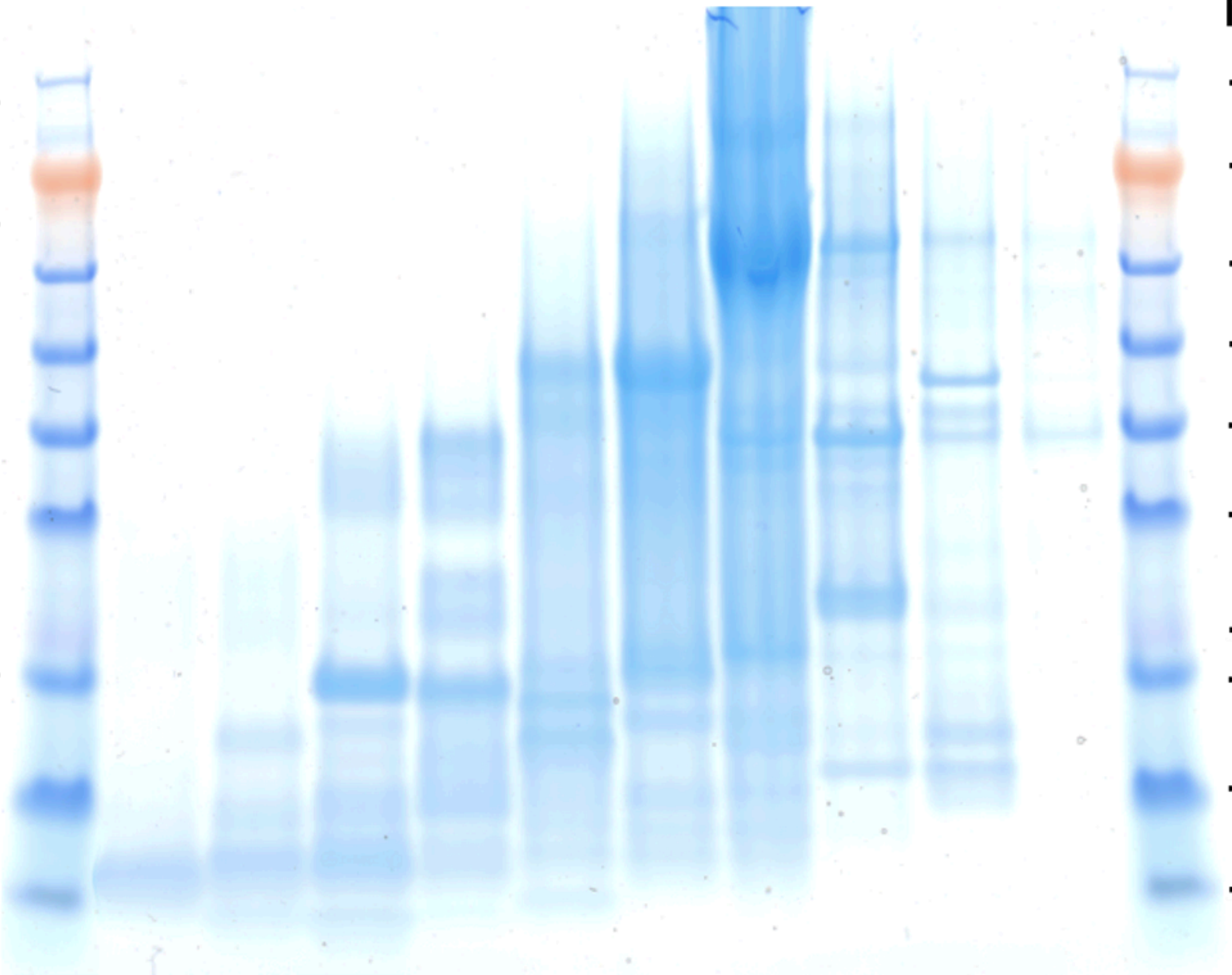

PAGE L52 HPLC Fractions		
L52 Lane	Fraction Number	Fig 5B Lane
1	SeeBlue+2	—
2	14	11
3	15	12
4	16	13
5	17	14
6	18	15
7	19	16
8	20	17
9	21	18
10	22	19
11	23	20
12	SeeBlue+2	—

Notes: Coomassie Blue stained PAGE gel. bLF standard molecular weights shown at left. SeeBlue standard weights at right. Table lists sample identities and lane order in Figure 5B.

PAGE L54 used in Figures 5C and 6

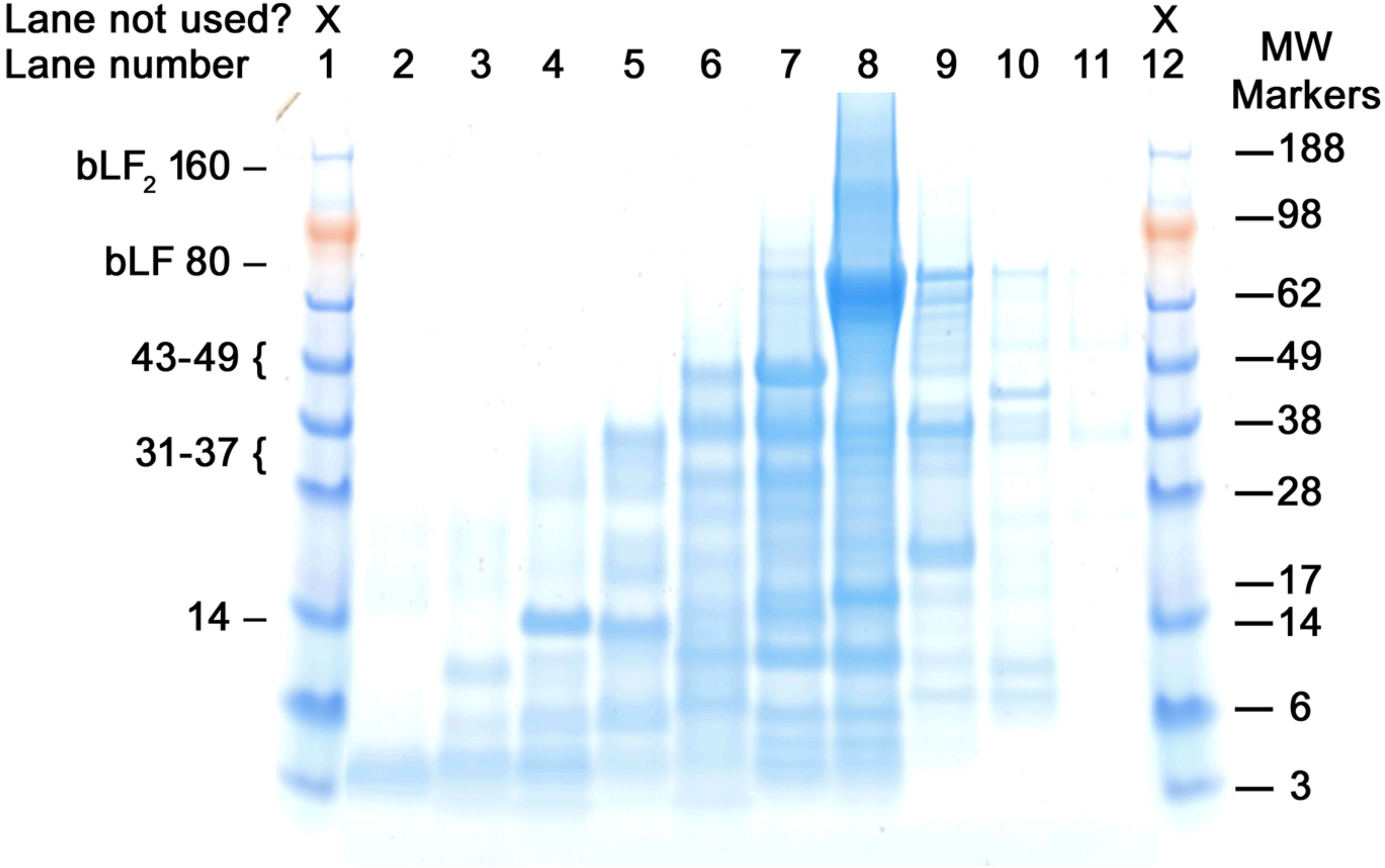

PAGE L54 HPLC Fractions			
L54 Lane	Fraction Number	Fig 5C Lane	Fig 6 Lane
1	SeeBlue+2	—	—
2	14	11	—
3	15	12	—
4	16	13	—
5	17	14	—
6	18	15	—
7	19	16	1
8	20	17	—
9	21	18	—
10	22	19	—
11	23	20	—
12	SeeBlue+2	—	—

Notes: Coomassie Blue stained PAGE gel. bLF standard molecular weights shown at left. SeeBlue standard weights at right. Table lists sample identities and lane order in Figures 5C and 6.

Western L55 used in Figure 5C

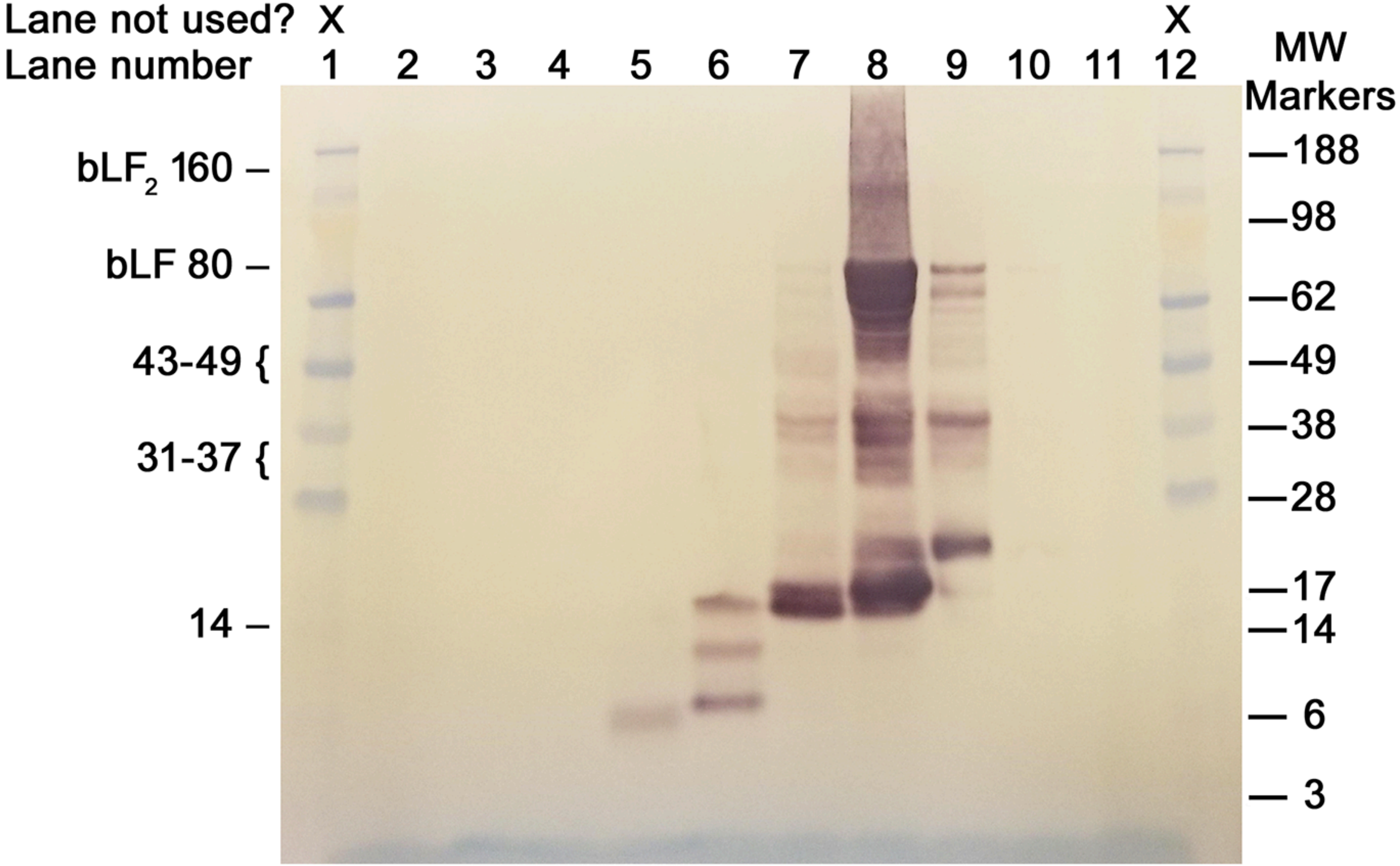

Western L55 HPLC Fractions		
L55 Lane	Fraction Number	Fig 5C Lane
1	SeeBlue+2	—
2	14	11
3	15	12
4	16	13
5	17	14
6	18	15
7	19	16
8	20	17
9	21	18
10	22	19
11	23	20
12	SeeBlue+2	—

Notes: Western blot developed with anti-N lobe mAb. bLF standard molecular weights shown at left. SeeBlue standard weights at right. Table lists sample identities and lane order in Figure 5C.

Western L56 used in Figure 5C

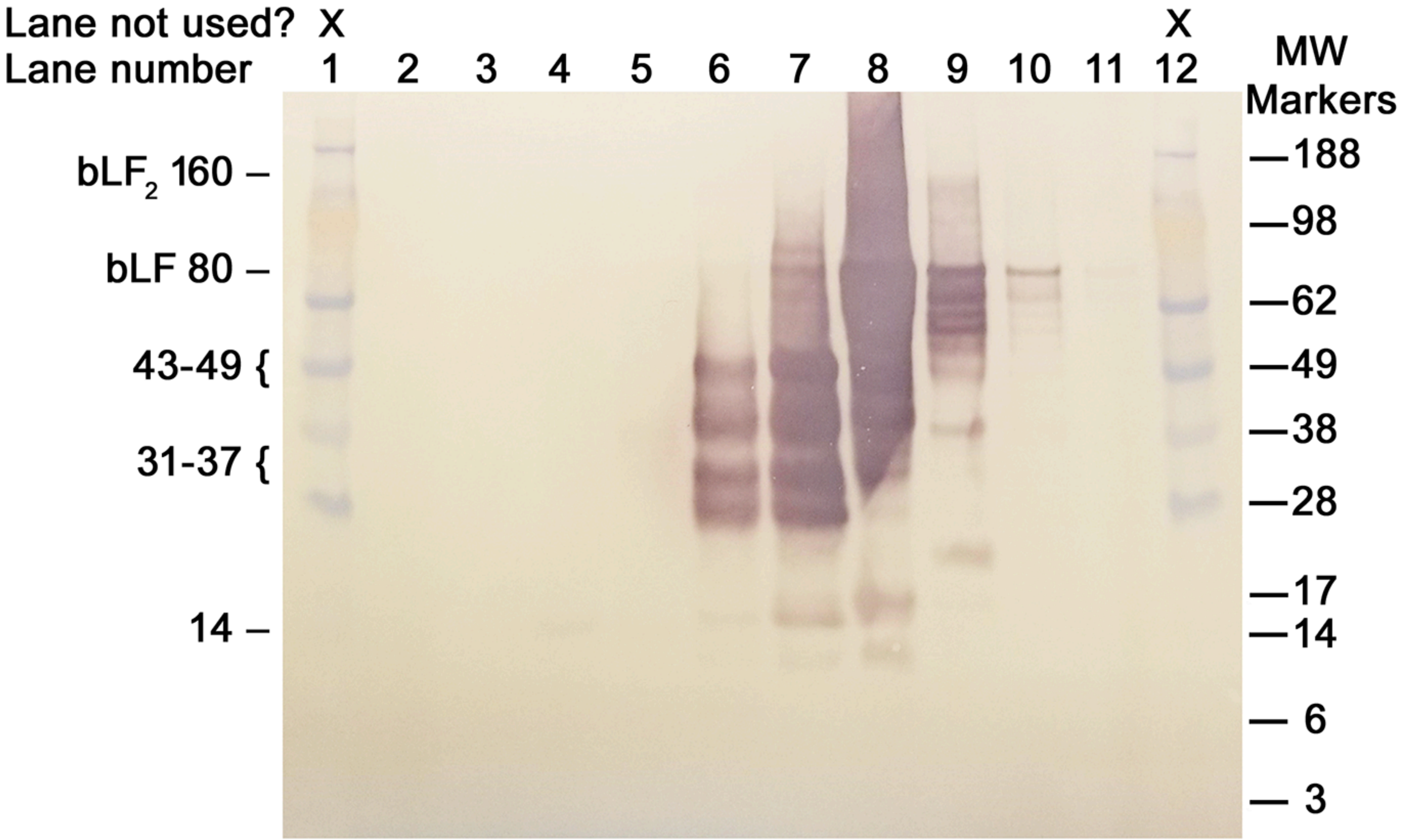

Western L56 HPLC Fractions		
L56 Lane	Fraction Number	Fig 5C Lane
1	SeeBlue+2	—
2	14	11
3	15	12
4	16	13
5	17	14
6	18	15
7	19	16
8	20	17
9	21	18
10	22	19
11	23	20
12	SeeBlue+2	—

Notes: Western blot developed with anti-C lobe mAb. bLF standard molecular weights shown at left. SeeBlue standard weights at right. Table lists sample identities and lane order in Figure 5C.

Western L57 used in Figure 5B

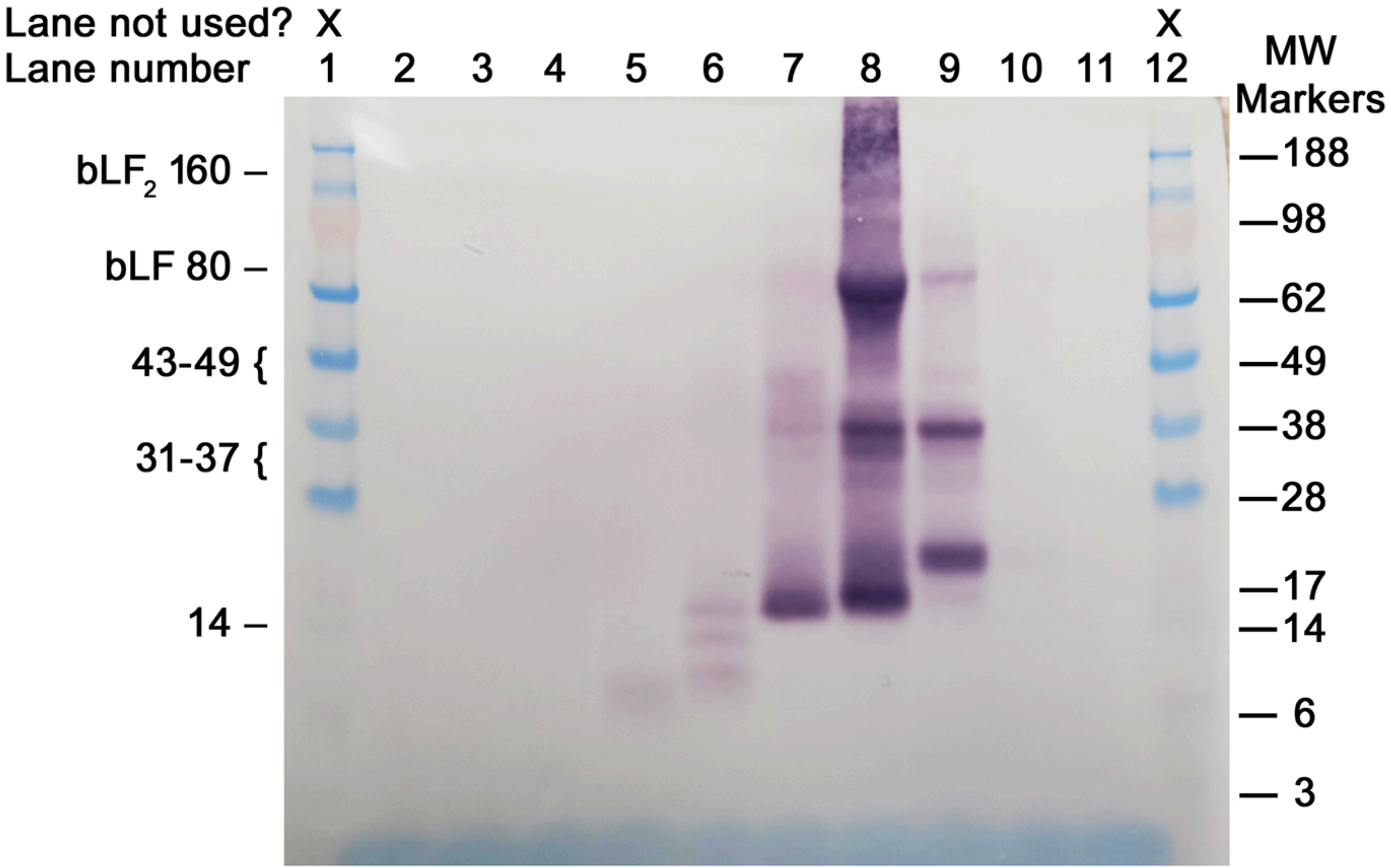

Western L57 HPLC Fractions		
L57 Lane	Fraction Number	Fig 5B Lane
1	SeeBlue+2	—
2	14	11
3	15	12
4	16	13
5	17	14
6	18	15
7	19	16
8	20	17
9	21	18
10	22	19
11	23	20
12	SeeBlue+2	—

Notes: Western blot developed with anti-N lobe mAb. bLF standard molecular weights shown at left. SeeBlue standard weights at right. Table lists sample identities and lane order in Figure 5B.

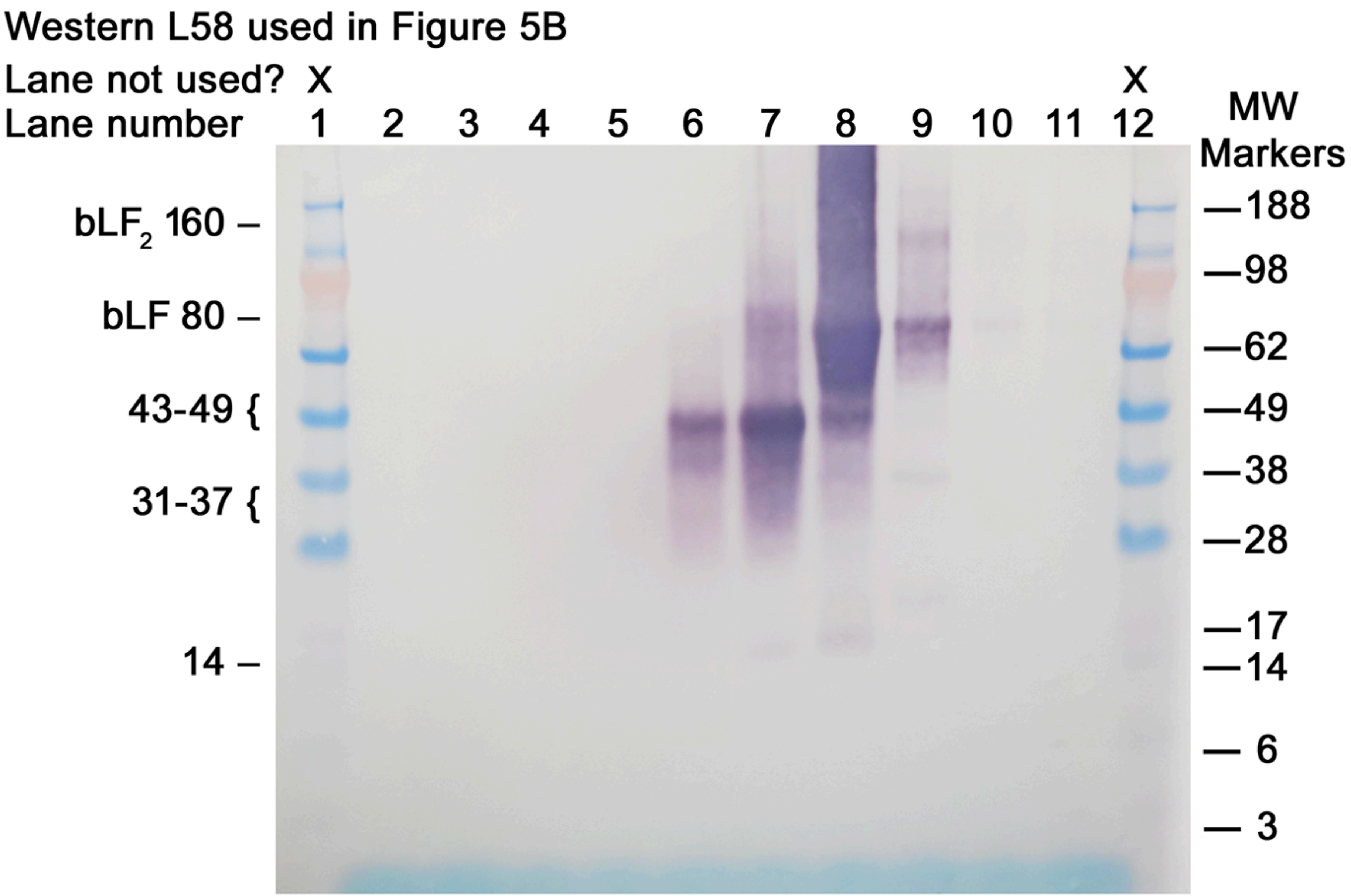

Western L58 HPLC Fractions		
L58 Lane	Fraction Number	Fig 5B Lane
1	SeeBlue+2	—
2	14	11
3	15	12
4	16	13
5	17	14
6	18	15
7	19	16
8	20	17
9	21	18
10	22	19
11	23	20
12	SeeBlue+2	—

Notes: Western blot developed with anti-C lobe mAb. bLF standard molecular weights shown at left. SeeBlue standard weights at right. Table lists sample identities and lane order in Figure 5B.

Western L59 used in Figure 5B

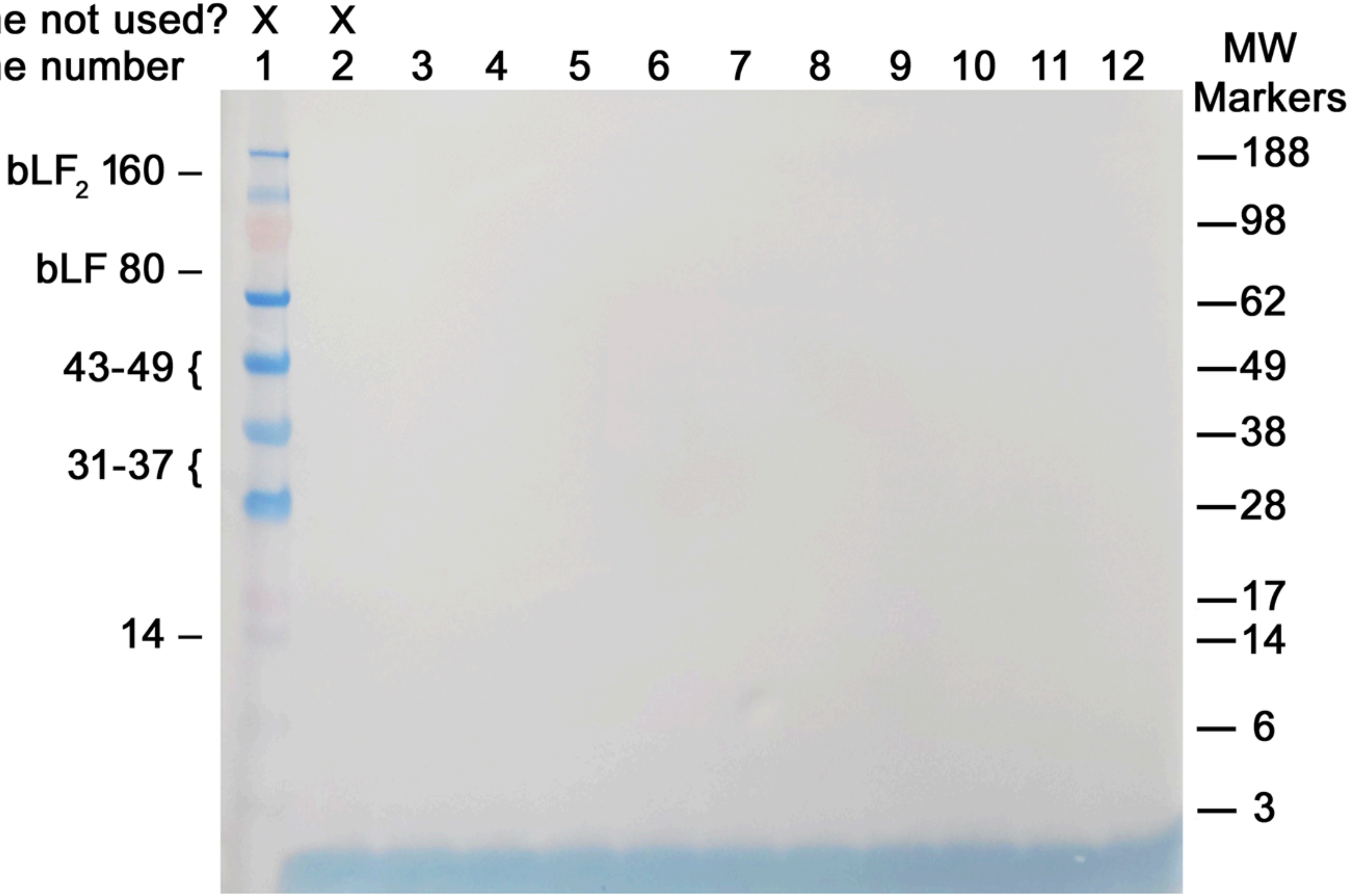

Western L59 HPLC Fractions		
L59 Lane	Fraction Number	Fig 5B Lane
1	SeeBlue+2	—
2	3	—
3	4	1
4	5	2
5	6	3
6	7	4
7	8	5
8	9	6
9	10	7
10	11	8
11	12	9
12	13	10

Notes: Western blot developed with anti-N lobe mAb. bLF standard molecular weights shown at left. SeeBlue standard weights at right. Table lists sample identities and lane order in Figure 5B.

Western L60 used in Figure 5B

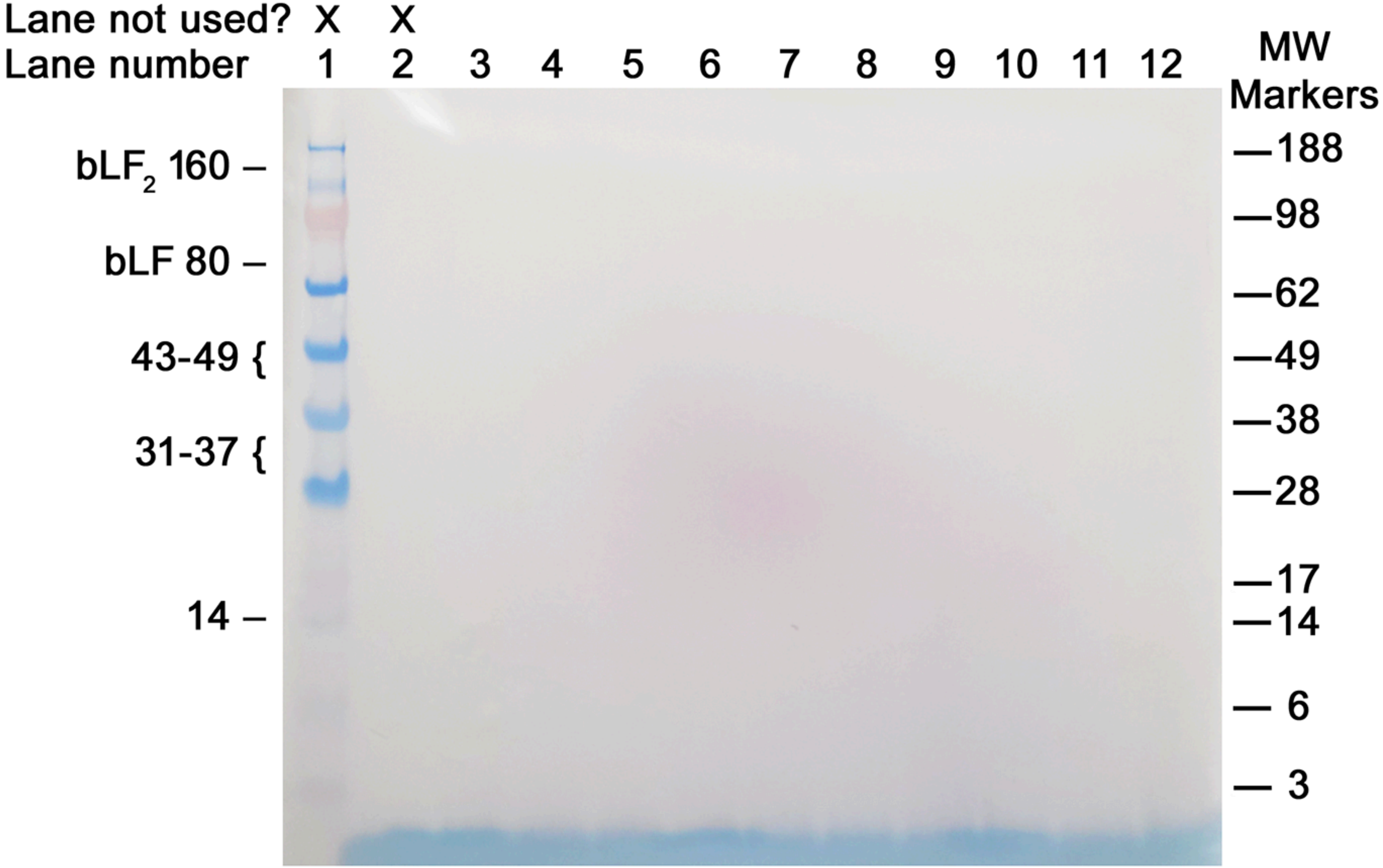

Western L60 HPLC Fractions		
L60 Lane	Fraction Number	Fig 5B Lane
1	SeeBlue+2	—
2	3	—
3	4	1
4	5	2
5	6	3
6	7	4
7	8	5
8	9	6
9	10	7
10	11	8
11	12	9
12	13	10

Notes: Western blot developed with anti-C lobe mAb. bLF standard molecular weights shown at left. SeeBlue standard weights at right. Table lists sample identities and lane order in Figure 5B.
