# Supplementary figures and images for: Characterization of proteolytic degradation products of vaginally administered bovine lactoferrin
Source: PLoS One. 2022 May 19;17(5):e0268537. doi: 10.1371/journal.pone.0268537 (PMC9119511; doi:10.1371/journal.pone.0268537)

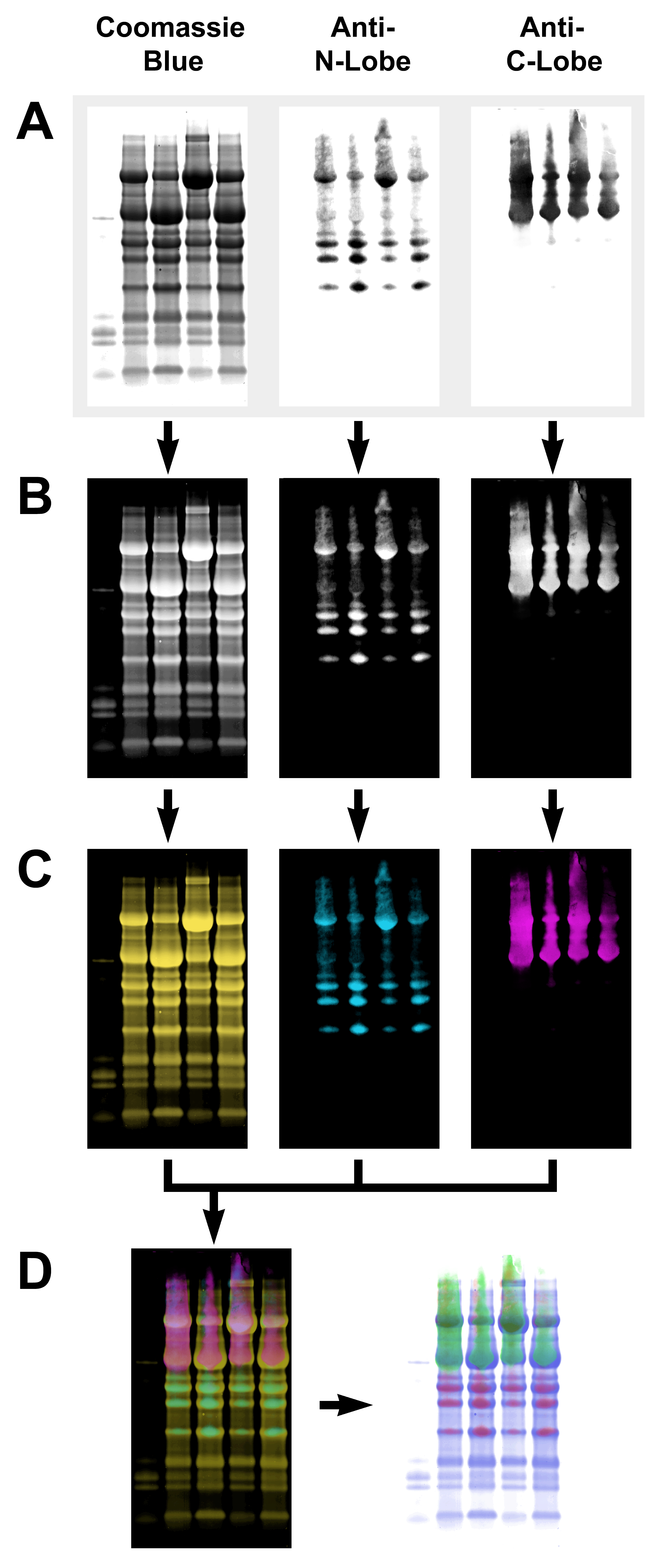

Supplement: S2 Fig — In panel A, selected lanes from PAGE Au26 (lanes 5–9, Coomassie blue stained) western blot Au38 (lanes 8–12, N-lobe mAb stained) and western blot Au37 (lanes 8–12, C-lobe mAb stained) are shown after being converted to black-and-white in Photoshop. In panel B, the images were inverted to black backgrounds and white-toned bands. In panel C, the images were converted to negative monochrome colors using Photoshop’s photo filter. In panel D, the three monochrome images were superimposed (left) and then inverted for the final composite tricolor image (right). Details of this procedure are given in the Methods section. (TIF) [file pone.0268537.s002.tif]
